# The CDK4/6 inhibitor dalpiciclib augments the antitumor efficacy of enzalutamide in preclinical models of castration-resistant prostate cancer through inhibition of MCM4-mediated DNA replication

**DOI:** 10.1038/s41419-026-08959-9

**Published:** 2026-06-05

**Authors:** Yue Liu, Yuan Ke, Jingtian Yu, Mengting Chai, Linhui Zheng, Chunqian Yang, Dong Liu, Shiyu Cheng, Zhiyuan Xiong, Jialiang Feng, Zhenpeng Zhao, Chaoyan Wu, Haijun Yu

**Affiliations:** 1 Department of Radiation and Medical Oncology, Zhongnan Hospital of Wuhan University, Wuhan, Hubei China; 2 Department of Integrated Traditional Chinese Medicine and Western Medicine, Zhongnan Hospital of Wuhan University, Wuhan, Hubei China; 3 Hubei Key Laboratory of Tumor Biological Behaviors, Wuhan, Hubei China

**Keywords:** Drug development, Translational research

## Abstract

Castration-resistant prostate cancer (CRPC) is a fatal malignancy often associated with alterations in cell cycle regulation, particularly within the Cyclin/CDK/RB axis. Despite ongoing clinical trials assessing CDK4/6 inhibitors in prostate cancer, clinical evidence remains limited. Although the androgen receptor (AR) inhibitor enzalutamide (ENZ) initially demonstrates therapeutic efficacy, resistance develops over time, and monotherapy offers limited antitumor benefits, highlighting the need for effective combination therapies. In this study, pharmacological profiling, genetic dependency analysis, RNA sequencing, and functional validation were conducted across various in vitro and in vivo preclinical CRPC models. The combination of ENZ and the CDK4/6 inhibitor dalpiciclib (DAL) exhibited potent antitumor activity in CRPC cell lines, a cell-derived xenograft (CDX) model, and a lymphatic metastatic model. Moreover, ENZ plus DAL inhibited cell cycle progression, migration, and DNA replication, while promoting apoptosis in CRPC cells. Mechanistically, ENZ blocked AR-mediated transcriptional activation of MCM4, a critical component of the DNA helicase complex, thereby enhancing the effect of CDK4/6 inhibition on DNA replication and inducing a pronounced synergistic antitumor response. These results suggest that the ENZ-DAL combination is a promising therapeutic approach that warrants further clinical evaluation in CRPC patients.

## Introduction

Prostate cancer (PCa) remains a leading cause of cancer-related mortality among men globally [1]. Androgen deprivation therapy (ADT) serves as the first-line treatment to inhibit PCa progression. While 80–90% of patients initially respond to ADT [2], nearly all eventually develop castration-resistant prostate cancer (CRPC), typically within three years of treatment initiation [3–5].

Enzalutamide (ENZ), a second-generation androgen receptor (AR) antagonist approved by the U.S. Food and Drug Administration (FDA), targets androgen signaling in CRPC patients [6]. By inhibiting AR nuclear translocation, DNA binding, and coactivator recruitment, ENZ suppresses CRPC cell proliferation and induces apoptosis. However, resistance inevitably arises through complex mechanisms, driving disease progression [7]. Therefore, rational combination strategies are necessary to enhance the efficacy of ENZ and delay the onset of resistance in CRPC.

Dysregulation of the Cyclin/CDK/RB axis, leading to aberrant activation of the cell cycle, is one of the most frequently altered pathways in PCa. This disruption results in uncontrolled G1/S phase transition, making the pathway an attractive therapeutic target [8]. Androgens act as mitogenic signals by promoting Cyclin D and CDK expression while inhibiting CDK inhibitors. Additionally, AR regulates the transcription of cell cycle-related genes, including Cyclin D [9]. CDK4/6 inhibitors block the phosphorylation of retinoblastoma (RB) protein by the Cyclin D-CDK4/6 complex, preventing E2F transcription factor release and inhibiting the G1/S transition. These findings suggest a functional link between AR signaling and cell-cycle progression, providing a rationale for combining CDK4/6 inhibition with ENZ in CRPC treatment.

Dalpiciclib (DAL), a selective CDK4/6 inhibitor approved in China for HR-positive, HER2-negative advanced breast cancer in combination with fulvestrant, has shown promising clinical efficacy in breast cancer [10]. Although CDK4/6 inhibitors have demonstrated preliminary activity in PCa [11], their therapeutic potential—particularly that of DAL, whether as monotherapy or in combination with other treatments—remains underexplored. Several clinical trials are currently evaluating CDK4/6 inhibitors in PCa, either alone or in combination with AR-targeted therapies. However, clinical evidence remains limited, and their therapeutic benefit has not yet been conclusively established. Therefore, this study aims to conduct a comprehensive preclinical evaluation of CDK4/6 inhibitors in combination with AR-targeted therapy in CRPC, offering a foundation for future therapeutic strategies.

This study integrated pharmacological profiling, pan-cancer genetic dependency analysis, RNA sequencing, and experimental validation across multiple in vitro and in vivo CRPC models to assess the therapeutic potential of DAL alone and in combination with ENZ. Additionally, the underlying molecular mechanisms were explored.

## Results

### CDK4 is upregulated and associated with poor clinical outcomes in CRPC

**Fig. 1:**
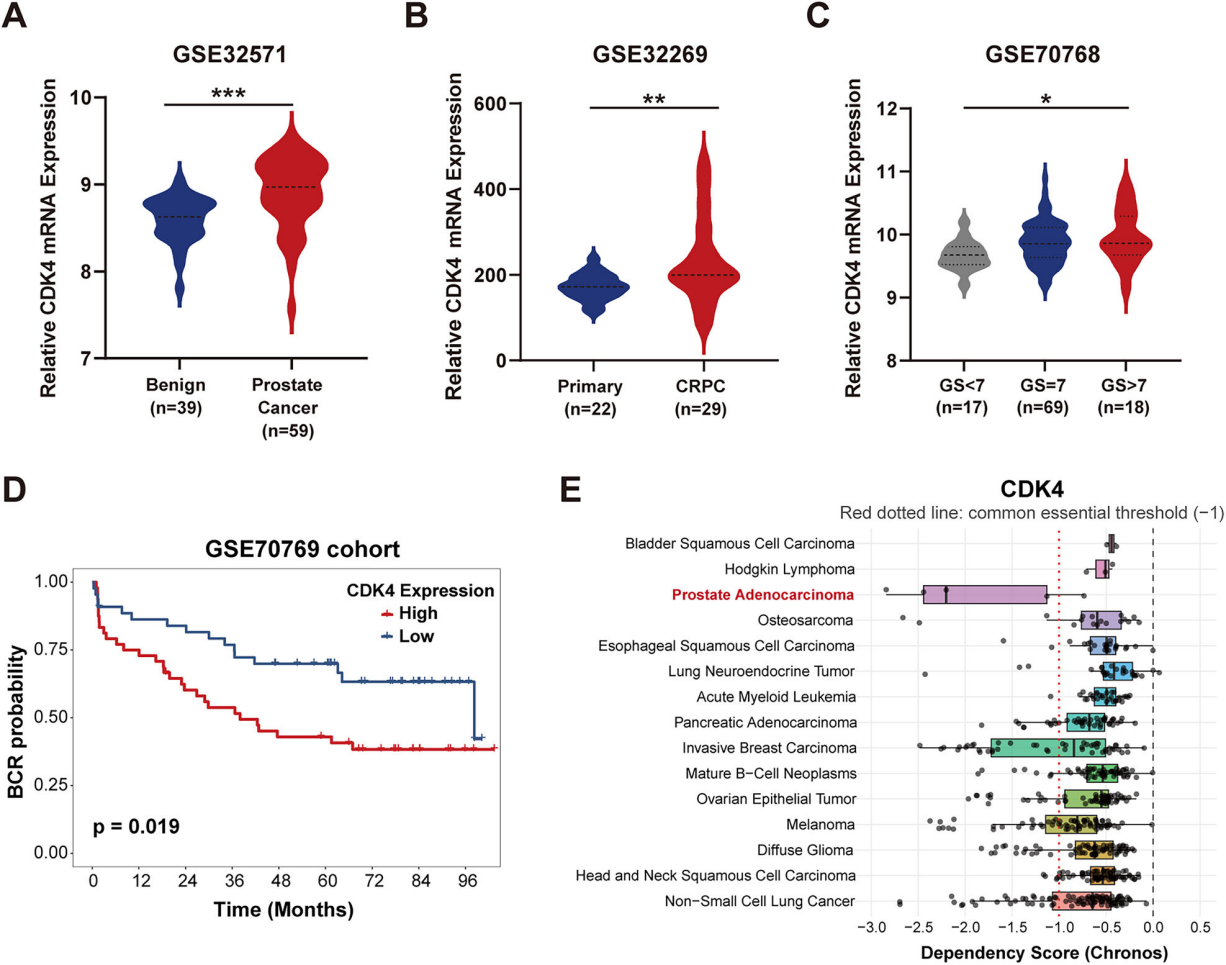
Elevated CDK4 expression correlates with prostate cancer progression and poor prognosis. A–C, CDK4 mRNA expression in prostate cancer patient cohorts from the GSE32571, GSE32269, and GSE70768 datasets. **A** CDK4 expression in benign prostate tissues and prostate cancer samples from the GSE32571 dataset. **B** CDK4 expression in primary prostate cancer and CRPC in the GSE32269 dataset. **C** CDK4 expression in prostate cancer samples stratified by Gleason score (GS<7, GS = 7, and GS>7) in the GSE70768 dataset. **D** Kaplan–Meier survival analysis of biochemical recurrence (BCR)-free survival in the GSE70769 cohort (RNA-seq data). Patients were stratified into CDK4-high (*n* = 48) and CDK4-low (*n* = 44) groups according to the optimal cutoff value of CDK4 expression. **E** CDK4 gene dependency scores across multiple cancer types based on CRISPR screening data from the DepMap Public 25Q2 dataset (Chronos algorithm). Thick vertical lines indicate median dependency scores (Prostate Adenocarcinoma, *n* = 5, median = −2.20). Dependency scores of 0 and −1 represent the medians of non-essential and common essential genes, respectively. Statistical significance was determined using a two-tailed unpaired Student’s *t*-test (**A**, **B**) or one-way ANOVA with Tukey’s multiple comparisons test (**C**). Survival differences were analyzed using the log-rank test (**D**). *p < 0.05, **p < 0.01, ***p < 0.001.

Given the frequent dysregulation of the Cyclin/CDK/RB axis in PCa and its functional association with AR-driven cell-cycle progression, the expression patterns and clinical relevance of CDK4 and CDK6 were first investigated across multiple public PCa transcriptomic datasets. Analysis of CDK4 expression at the mRNA level in the GSE32571 dataset revealed significant upregulation of CDK4 in prostate tumor tissues compared to adjacent benign prostate tissues (Fig. 1A). Similarly, in the GSE32269 dataset, CDK4 expression was markedly higher in CRPC specimens than in localized prostate tumors (Fig. 1B). In contrast, CDK6 expression did not show a statistically significant difference (Supplementary Fig. S1A, B). Moreover, in the GSE70768 dataset, patients with high Gleason scores (GS>7) exhibited significantly elevated CDK4 mRNA levels compared to those with low GS (<7) (Fig. 1C). Kaplan–Meier survival analysis of the GSE70769 cohort revealed that higher CDK4 mRNA expression was associated with a reduced probability of biochemical recurrence-free (BCR) survival (*p* = 0.019), indicating that elevated CDK4 expression correlates with an unfavorable clinical outcome (Fig. 1D).

Additionally, a pan-cancer dependency analysis was performed using the DepMap Public 25Q2 dataset to assess CDK4’s essentiality. The Chronos algorithm identified a marked dependency on CDK4 in prostate adenocarcinoma cell lines, with a median gene effect score of –2.20 (ranging from –2.84 to –0.735), substantially lower than the common essentiality threshold of –1 (Fig. 1E). These findings suggest that CDK4 is not only associated with adverse clinical features but may also represent a potential therapeutic vulnerability in PCa. These results highlight CDK4 as a clinically relevant and functionally significant target in CRPC.

### CDK4/6 inhibitor dalpiciclib synergizes with enzalutamide to suppress CRPC cell growth

**Fig. 2:**
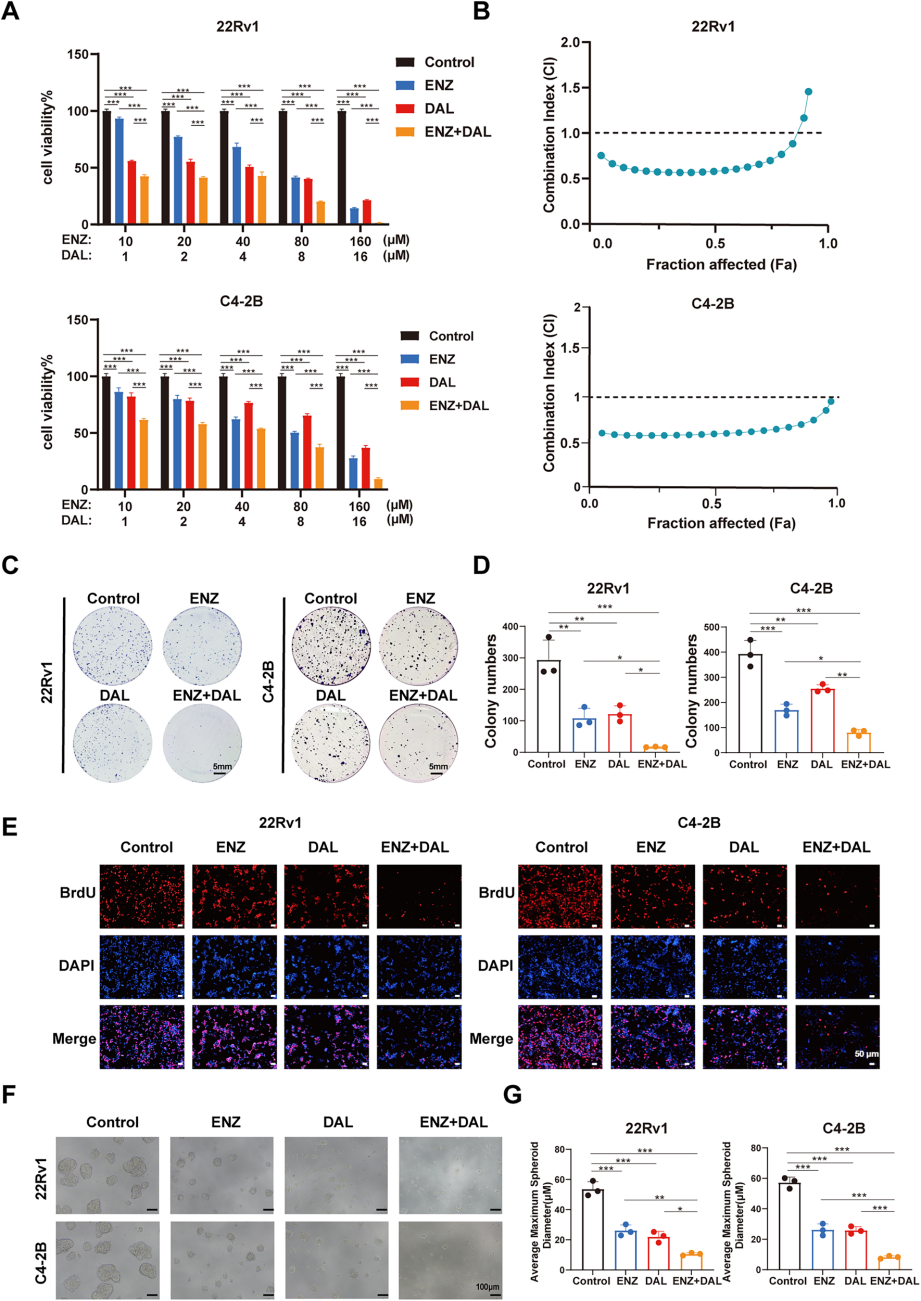
Synergistic inhibition of CRPC cell growth by the CDK4/6 inhibitor dalpiciclib in combination with enzalutamide. **A** 22Rv1 and C4-2B cells were treated for 48 h with ENZ and DAL in a fixed-ratio combination (10:1), with 2-fold serial dilutions of ENZ (10–160 μM) and DAL (1–16 μM). **B** Combination index (CI) values were calculated using the Chou-Talalay method based on cell viability data. CI values < 1, = 1, and >1 indicate synergy, an additive effect, and antagonism, respectively. **C** Representative images of colony formation assays in 22Rv1 and C4-2B cells treated with Control (DMSO), ENZ (35 μM), DAL (2 μM), or their combination for 12–17 days (scale bar = 5 mm). **D** Quantification of colony numbers from colony formation assays. **E** Representative immunofluorescence images of BrdU incorporation assays in 22Rv1 and C4-2B cells treated with Control (DMSO), ENZ (35 μM), DAL (2 μM), or their combination for 48 h (scale bar = 50 μm). **F** Representative images from 3D spheroid formation assays in 22Rv1 and C4-2B cells cultured in ultra-low attachment 96-well plates and treated with Control (DMSO), ENZ (35 μM), DAL (2 μM), or the combination for 7 days (scale bar = 100 μm). **G** Quantitative analysis of the average maximum diameter of 3D spheroids across different treatment groups. Statistical significance was determined by one-way ANOVA with Tukey’s multiple comparisons test (**A**, **D**, **G**). For **A**, statistical comparisons were performed among treatment groups at each dose level. Quantitative data are presented as mean ± SD from three independent experiments. *p < 0.05, **p < 0.01, ***p < 0.001.

Given the potential therapeutic relevance of the Cyclin/CDK/RB axis in CRPC, this study further investigated whether pharmacological CDK4/6 inhibition with DAL could enhance the antitumor activity of ENZ. First, half-maximal inhibitory concentrations (IC_50_) of DAL and ENZ were determined in two CRPC cell lines, 22Rv1 and C4-2B, using dose–response assays. In 22Rv1 cells, the median IC_50_ of ENZ was 51.72 μM (95% CI: 47.58–56.35 μM), while for DAL, it was 7.621 μM (95% CI: 6.345–9.469 μM). In C4-2B cells, the IC_50_ values were 70.53 μM (95% CI: 64.30–77.67 μM) for ENZ and 3.729 μM (95% CI: 3.403–4.084 μM) for DAL (Supplementary Fig. S2A–D). Cells were then treated with a series of ENZ:DAL combinations at a fixed ratio of 10:1 for 48 h, followed by cell viability assessment. The combination treatment resulted in significantly greater inhibition of CRPC cell growth compared to either agent alone or vehicle control (Fig. 2A). To further assess the interaction between the two agents, synergy analysis was performed using the CompuSyn model. Increasing concentrations of ENZ and DAL enhanced growth suppression in both cell lines, with combination index (CI) values consistently < 1, indicating a synergistic effect (Fig. 2B).

To further evaluate the pharmacological efficacy of the ENZ-DAL combination, colony formation assays were conducted. Compared to monotherapy, the combination treatment markedly suppressed the clonogenic potential of CRPC cells (Fig. 2C, D). BrdU immunofluorescence staining confirmed that co-treatment with ENZ and DAL significantly inhibited DNA replication and reduced proliferative activity (Fig. 2E). Since three-dimensional (3D) culture systems better replicate the tumor microenvironment than conventional two-dimensional (2D) models, a 3D tumor spheroid assay was utilized to assess treatment response. The combination significantly reduced the size of 3D spheroids formed by 22Rv1 and C4-2B cells, showing stronger inhibitory effects than either agent alone (Fig. 2F, G). These results demonstrate that DAL synergizes with ENZ to effectively suppress CRPC cell growth and proliferation.

### Combination of the CDK4/6 inhibitor dalpiciclib and enzalutamide synergistically induces apoptosis and inhibits cell cycle progression in CRPC cells

**Fig. 3:**
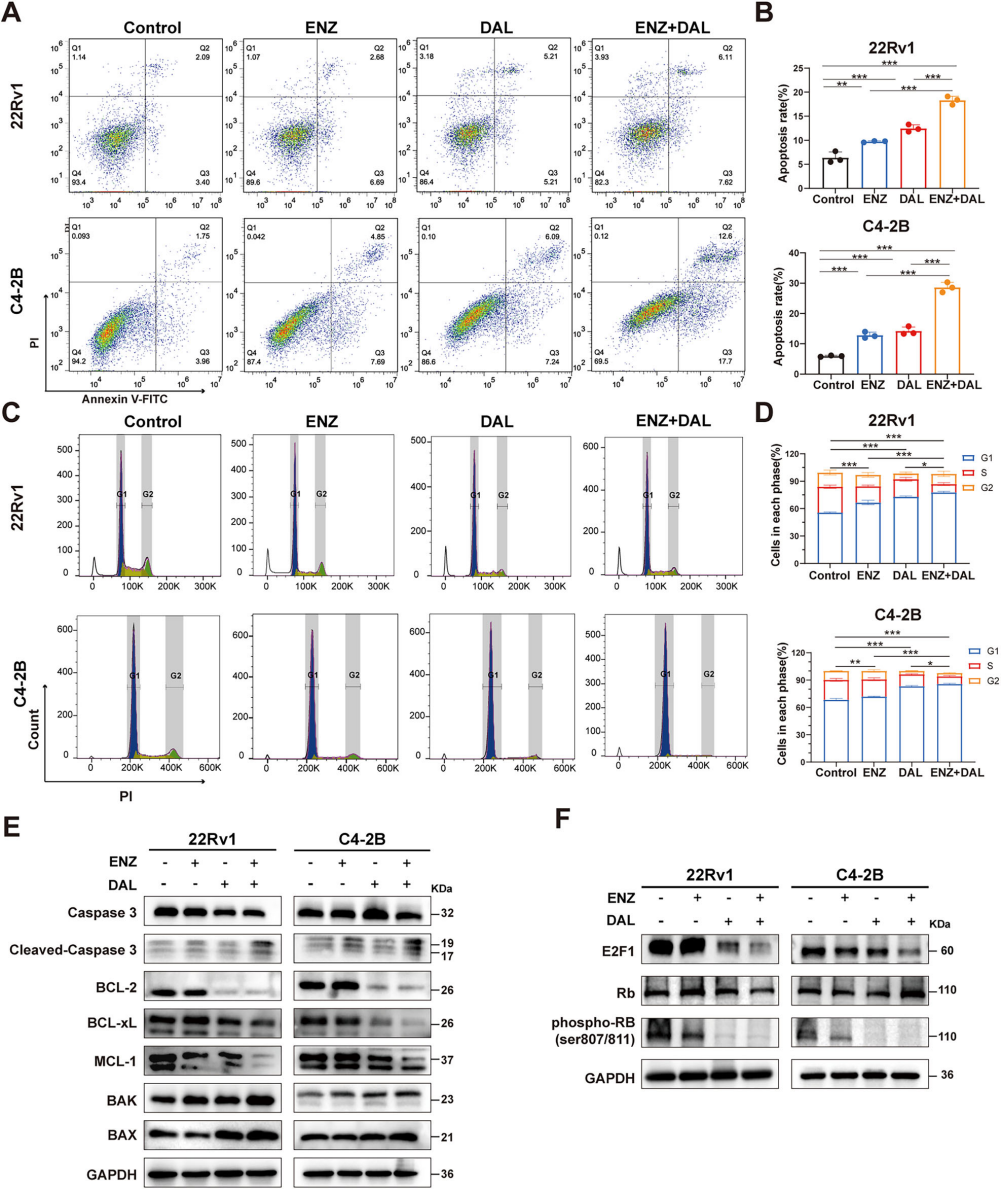
Dalpiciclib enhances apoptosis and induces cell cycle arrest in CRPC cells when combined with enzalutamide. **A** Representative Annexin V/PI flow cytometry plots showing apoptosis in 22Rv1 and C4-2B cells after 48-hour treatment with Control (DMSO), ENZ (35 μM), DAL (2 μM), or their combination. **B** Quantification of total apoptotic cells (early + late apoptosis) in 22Rv1 and C4-2B cells under the indicated treatments. **C** Representative flow cytometry histograms showing cell cycle distribution in 22Rv1 and C4-2B cells after 48-hour treatment with Control (DMSO), ENZ (35 μM), DAL (2 μM), or their combination. **D** Quantification of cell cycle distribution, showing the percentages of cells in G1, S, and G2/M phases. Statistically significant differences in the G1-phase fraction between the indicated groups are indicated by asterisks. **E** Western blot analysis of apoptosis-related proteins in 22Rv1 and C4-2B cells treated with or without ENZ (35 μM) and/or DAL (2 μM) for 48 h. **F** Western blot analysis of cell cycle-related proteins in 22Rv1 and C4-2B cells treated with or without ENZ (35 μM) and/or DAL (2 μM) for 48 h. Statistical significance was determined by one-way ANOVA with Tukey’s multiple comparisons test (B, D). Quantitative data are presented as mean ± SD from three independent experiments. *p < 0.05, **p < 0.01, ***p < 0.001.

Apoptosis is a key mechanism of cancer cell death. To assess whether the combination of ENZ and the CDK4/6 inhibitor DAL enhances apoptosis in CRPC cells, flow cytometric analysis was performed using Annexin V/PI staining. The ENZ + DAL combination significantly increased the proportion of apoptotic cells compared to either monotherapy (Fig. 3A, B). Next, the effects of these treatments on cell cycle progression were analyzed. Flow cytometry showed that DAL monotherapy induced a substantial G1-phase arrest, whereas ENZ alone caused only a modest accumulation in G1. Notably, the ENZ + DAL combination resulted in a marked increase in the G1 population, indicating that ENZ sensitizes CRPC cells to DAL-induced cell cycle blockade (Fig. 3C, D). To elucidate the molecular mechanisms underlying the enhanced apoptotic response, key regulators of the intrinsic apoptotic pathway, including caspase activation and members of the BCL-2 protein family, were examined. Immunoblotting revealed that combined ENZ and DAL treatment led to a more significant increase in cleaved caspase-3 compared to either monotherapy. Additionally, there was a shift in the balance of BCL-2 family proteins toward a pro-apoptotic state, characterized by upregulation of the pro-apoptotic effectors BAX and BAK, alongside a marked reduction in the anti-apoptotic proteins BCL-2, BCL-xL, and MCL-1 (Fig. 3E). These changes support enhanced mitochondrial apoptotic priming upon dual AR and CDK4/6 inhibition. Consistent with CDK4/6 inhibition, the combination treatment significantly reduced phosphorylation of the RB protein and downregulated E2F1 expression (Fig. 3F).

In summary, these results demonstrate that the combination of ENZ and DAL synergistically inhibits CRPC progression by promoting apoptosis and inducing G1-phase cell cycle arrest.

### Combination therapy with enzalutamide and dalpiciclib suppresses CRPC metastatic potential

**Fig. 4:**
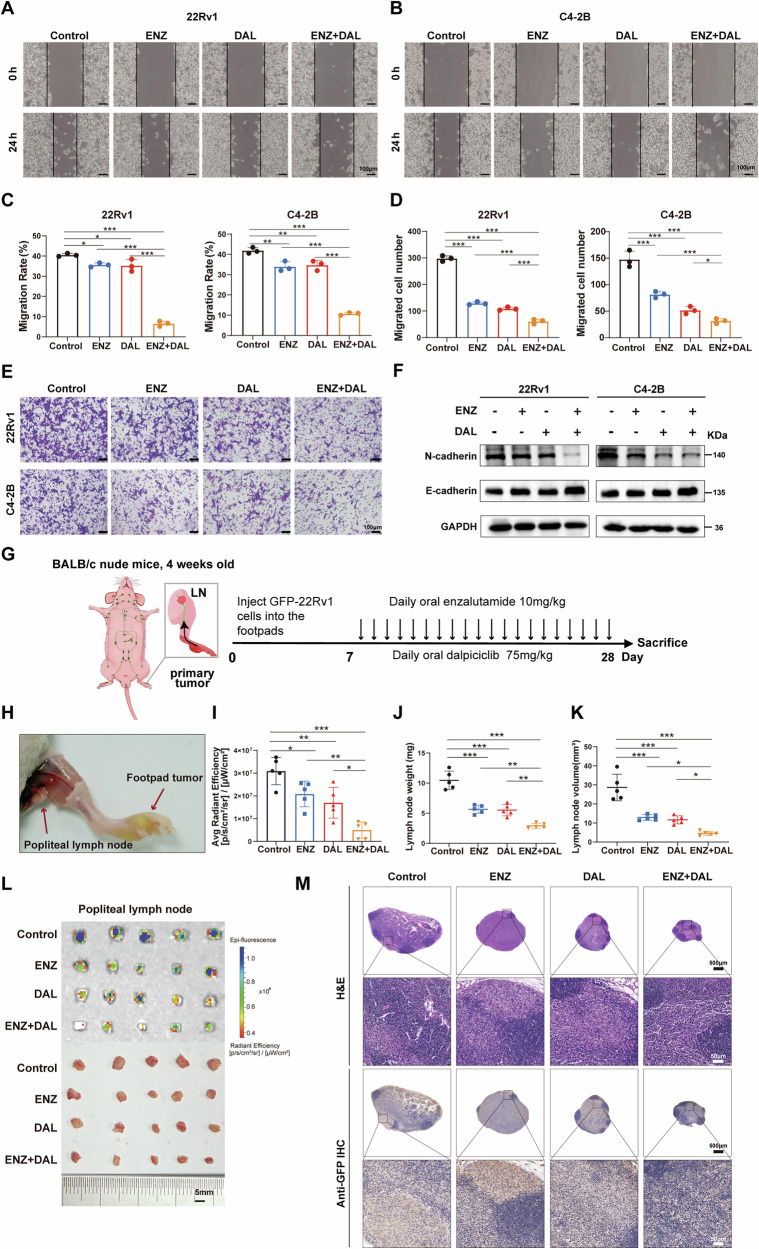
Synergistic suppression of CRPC metastatic potential by combined CDK4/6 inhibition and enzalutamide treatment. Representative images of wound-healing assays conducted on 22Rv1 (**A**) and C4-2B (**B**) cells treated with DMSO, ENZ (35 μM), DAL (2 μM), or ENZ (35 μM) + DAL (2 μM) for 24 h (scale bar = 100 μm). **C** Quantitative analysis of wound closure in 22Rv1 and C4-2B cells from three independent experiments. **D** Quantification of migrated cells from Transwell assays. Cells were treated with DMSO, ENZ (35 μM), DAL (2 μM), or ENZ (35 μM) + DAL (2 μM) for 48 h. **E** Representative images from Transwell migration assays using 22Rv1 and C4-2B cells (scale bar = 100 μm). **F** Western blot analysis of EMT-related proteins (E-cadherin and N-cadherin) in 22Rv1 and C4-2B cells treated with Control (DMSO), ENZ (35 μM), DAL (2 μM), or their combination for 48 h. **G** Schematic illustration of the popliteal lymph node metastasis model and treatment schedule in BALB/c nude mice. **H** Representative image showing the footpad tumor and popliteal lymph node in a nude mouse. **I** Quantification of bioluminescence signal intensity in popliteal lymph nodes (*n* = 5). **J** Quantification of popliteal lymph node weights at the endpoint (*n* = 5). **K** Quantification of popliteal lymph node volumes at the endpoint (*n* = 5). **L** Bioluminescence imaging and representative photographs of excised popliteal lymph nodes from nude mice at the experimental endpoint (day 28). **M** Representative images of H&E and anti-GFP IHC staining of popliteal lymph nodes from nude mice. Black scale bars, 500 μm; white scale bars, 50 μm. Statistical significance was determined by one-way ANOVA with Tukey’s multiple comparisons test (**C**, **D**, **I**–**K**). Quantitative data are presented as mean ± SD. *p < 0.05, **p < 0.01, ***p < 0.001.

The effects of ENZ and DAL on cell migration were assessed using wound-healing and Transwell assays. The combination treatment significantly reduced the migratory capacity of both 22Rv1 and C4-2B cells compared to either monotherapy (Fig. 4A–C). Similarly, transwell migration assays showed a notable decrease in migratory capacity in the ENZ + DAL group relative to ENZ or DAL alone (Fig. 4D, E). Since epithelial–mesenchymal transition (EMT) is a key process in tumor metastasis, the expression of EMT-related markers was examined by Western blot analysis. Co-treatment with ENZ and DAL led to upregulation of the epithelial marker E-cadherin and downregulation of the mesenchymal marker N-cadherin, indicating suppression of the EMT phenotype (Fig. 4F).

Lymph node (LN) metastasis is commonly observed in PCa and serves as a critical route for tumor spread. To further investigate the effect of ENZ + DAL combination therapy on CRPC LN metastasis in vivo, a mouse popliteal LN metastasis model was established by implanting GFP-labeled 22Rv1 cells into the footpads of BALB/c nude mice (*n* = 5 per group). One week after tumor implantation, mice were treated with ENZ and/or DAL via oral gavage daily for 21 days (Fig. 4G). Analysis of GFP fluorescence signals using the IVIS imaging system showed that the ENZ + DAL combination significantly reduced GFP fluorescence intensity in the popliteal LNs compared to the control, suggesting that the combination therapy inhibits CRPC cell metastasis to the popliteal LNs in nude mice. Consistently, both the volume and weight of the popliteal LNs in the combination group were significantly reduced compared to the control and monotherapy groups (Fig. 4H–L). Hematoxylin and eosin (H&E) and immunohistochemical (IHC) analysis further demonstrated a lower rate of LN metastasis in the combination group compared to the controls (Fig. 4M).

These results indicate that the combined ENZ and DAL treatment effectively suppresses the migratory capacities of CRPC cells in vitro and significantly inhibits LN metastasis in vivo, demonstrating a potent anti-metastatic effect.

### CDK4/6 inhibition specifically enhances the antitumor efficacy of AR blockade by suppressing DNA replication

**Fig. 5:**
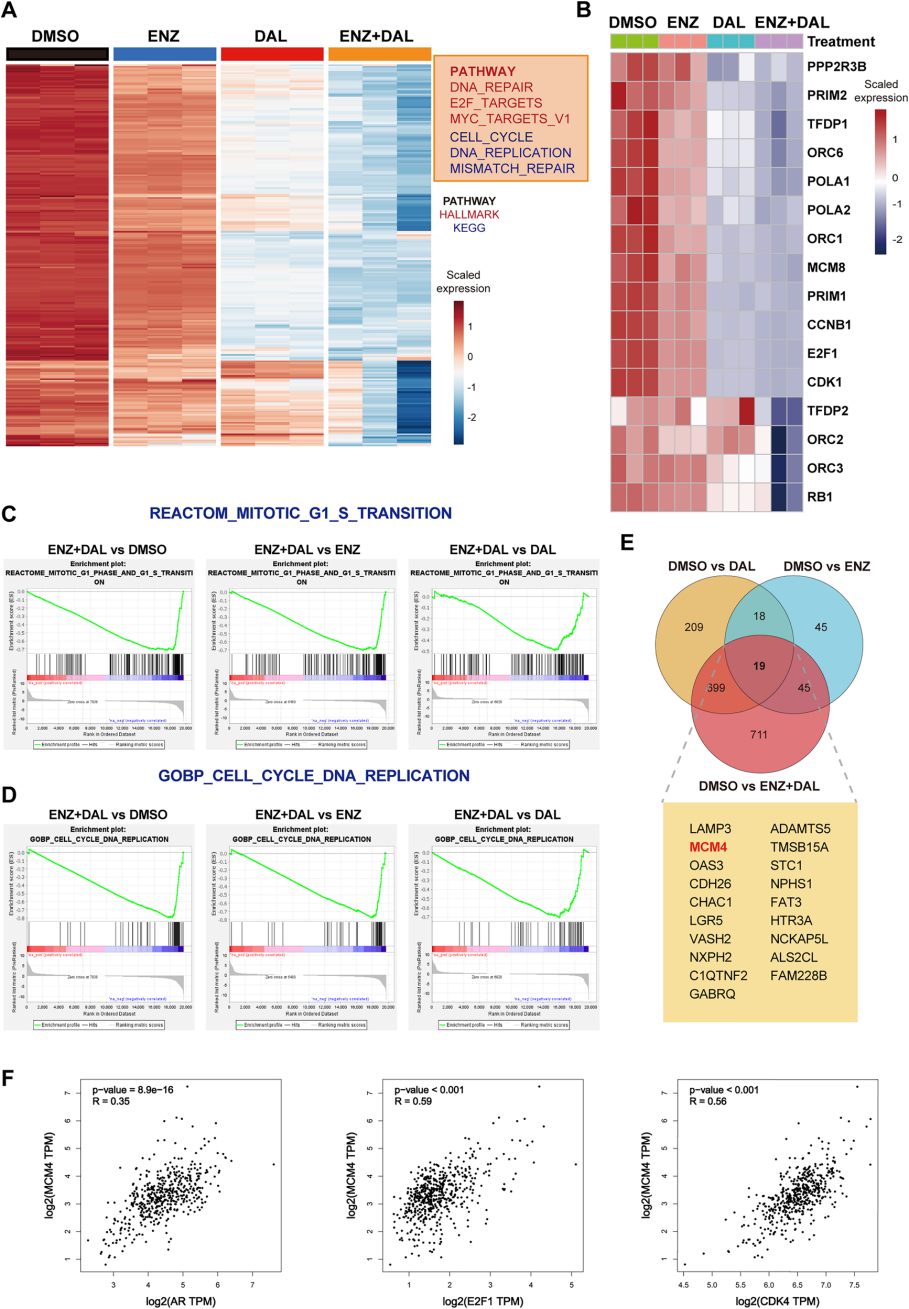
Enhanced efficacy of AR inhibitors by CDK4/6 inhibition through suppression of DNA replication. **A** Heatmap showing transcriptomic changes in 22Rv1 cells treated with DMSO, ENZ (35 μM), DAL (2 μM), or ENZ (35 μM) + DAL (2 μM) for 48 h. The pathway terms annotated on the right represent significantly enriched Hallmark and KEGG pathways identified from transcriptomic analysis; Hallmark pathways were derived from GSEA, whereas KEGG pathways were derived from enrichment analysis of differentially expressed genes. **B** Heatmap showing representative genes involved in DNA replication-related genes, including genes from the KEGG DNA replication pathway. **C**, **D** GSEA plots comparing the regulation of mitotic G1/S transition and DNA replication in the cell cycle between different treatments. **E** Venn diagram illustrating the overlap of DEGs between different treatment groups. **F** Correlation analysis of AR, E2F1, and CDK4 expression with MCM4 expression in the TCGA–PRAD cohort using the GEPIA.

To elucidate the synthetic lethal mechanism underlying the combination of ENZ and DAL, transcriptomic profiling was conducted on 22Rv1 cells treated with DMSO, ENZ, DAL, or the ENZ + DAL combination. To elucidate the molecular mechanisms underlying the synergistic effect, we identified differentially expressed genes (DEGs) between the ENZ + DAL combination and DMSO control groups (|LogFC | >1, *p* < 0.05). Reflecting the canonical pharmacological mechanisms of AR and CDK4/6 blockade, KEGG pathway enrichment analysis revealed that these DEGs were predominantly enriched in pathways associated with the cell cycle, DNA replication, and mismatch repair (Supplementary Fig. S3A). Gene Set Enrichment Analysis (GSEA) further corroborated these findings, demonstrating a significant downregulation of gene sets related to E2F targets, DNA replication, and cell cycle progression in the combination group (Supplementary Fig. S3B). Notably, while single agents showed partial effects, the suppressive impact on these core proliferative pathways was significantly intensified in the ENZ + DAL group (Fig. 5A). In particular, the expression of core genes within the DNA replication pathway—which exhibited the highest RichFactor values—was markedly inhibited following the combination treatment (Fig. 5B). The concurrent inhibition of both cell cycle progression and DNA replication was much more pronounced in cells exposed to ENZ + DAL compared to either monotherapy or the control group (Fig. 5C, D). These results suggest that AR blockade enhances the suppressive effect of CDK4/6 inhibition on DNA replication and cell cycle-related transcriptional programs.

To further elucidate the underlying mechanism, this study identified 19 commonly regulated genes from the intersection of DEG sets across the ENZ + DAL, ENZ, and DAL treatments relative to DMSO. Among these, MCM4—a key component of the DNA replication machinery—was significantly downregulated by the combination therapy, prompting us to focus on MCM4 as a critical candidate mediator of the synergistic effect (Fig. 5E). The minichromosome maintenance (MCM) protein family, particularly the MCM2-7 complex, plays a central role in replication licensing and cell cycle control [12]. Dysregulation of MCM proteins contributes to genomic instability and uncontrolled proliferation, which are hallmark features of malignant transformation. MCM4, in particular, functions as a core subunit of the replicative helicase complex and is essential for DNA unwinding at replication origins [13]. Mechanistically, the Cyclin D1-CDK4/6 complex promotes phosphorylation and inactivation of RB, leading to the activation of the transcription factor E2F1. E2F1 regulates the G1/S phase transition and drives the transcription of replication-associated genes, including MCM4 [14, 15]. Gene correlation analysis of the TCGA-PRAD dataset using GEPIA revealed strong positive correlations between CDK4, CDK6, E2F1, and MCM4 (Fig. 5F, Supplementary Fig. S3C). Interestingly, our analysis also revealed a positive correlation between AR and MCM4 expression (Fig. 5F), a previously unreported relationship.

These observations prompted further investigation into how AR regulates MCM4 expression and how the combination of ENZ and DAL synergizes to suppress DNA replication in CRPC cells.

### AR inhibition synergistically enhances the suppressive effect of CDK4/6 inhibition on DNA replication in CRPC cells through transcriptional repression of MCM4

**Fig. 6:**
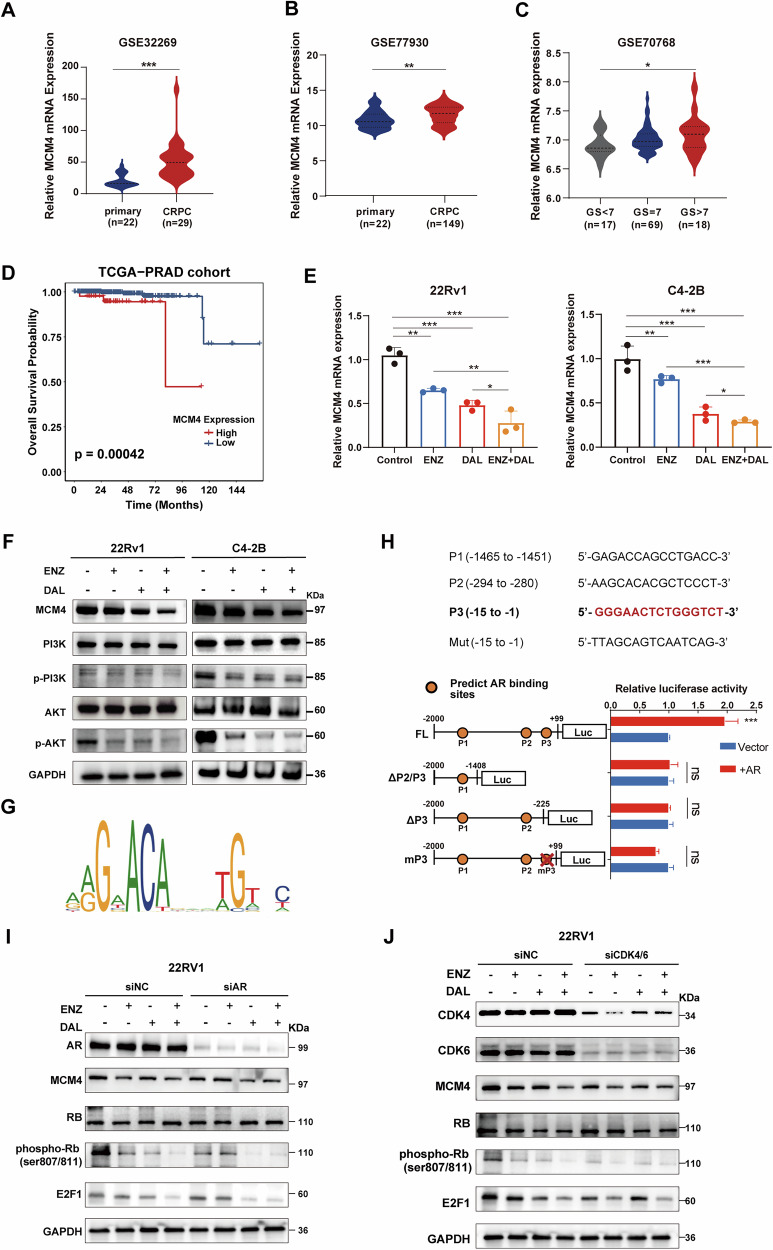
Synergistic enhancement of CDK4/6 inhibitor-induced suppression of DNA replication in CRPC cells by AR inhibition through transcriptional repression of MCM4. **A**–**C** MCM4 mRNA expression levels in patient cohorts from the GSE32269, GSE77930, and GSE70768 datasets. **D** Kaplan–Meier survival analysis of patients in the TCGA-PRAD cohort based on MCM4 mRNA expression levels. Patients were stratified into MCM4-high (*n* = 44) and MCM4-low (*n* = 392) groups according to the optimal cutoff value for MCM4 expression. Patients with missing survival data were excluded from the analysis. **E** qRT-PCR analysis of MCM4 mRNA expression in 22Rv1 and C4-2B cells treated with DMSO, ENZ (35 μM), DAL (2 μM), or their combination for 48 h. **F** Western blot analysis of MCM4 and PI3K/AKT signaling-related proteins in cells treated with DMSO, ENZ (35 μM), DAL (2 μM), or their combination for 48 h. **G** Consensus AR-binding motif obtained from the JASPAR database. **H** Predicted AR-binding sites in the MCM4 promoter, schematic diagrams of luciferase reporter constructs, and luciferase reporter assays in 293 T cells co-transfected with AR and the indicated MCM4 promoter constructs for 48 h (*n* = 3). **I** 22Rv1 cells transfected with control or AR-targeting siRNA were treated with DMSO, ENZ (35 μM), DAL (2 μM), or their combination for 48 h, followed by Western blot analysis of AR signaling, RB phosphorylation, and DNA replication proteins. **J** 22Rv1 cells transfected with control or CDK4/6-targeting siRNA were treated with DMSO, ENZ (35 μM), DAL (2 μM), or their combination for 48 h, followed by Western blot analysis of AR signaling, RB phosphorylation, and DNA replication proteins. Statistical significance was determined using a two-tailed unpaired Student’s *t*-test (**A**, **B**, **H**) or one-way ANOVA with Tukey’s multiple comparisons test (**C**, **E**). For **H**, statistical comparisons were made between Vector and +AR groups for each construct. Survival differences were analyzed using the log-rank test (**D**). Data are presented as mean ± SD. *p < 0.05, **p < 0.01, ***p < 0.001; ns, not significant.

Accumulating evidence suggests that dysregulated MCM4 expression is linked to tumorigenesis and poor clinical outcomes in various malignancies. Overexpression of MCM4 has been associated with enhanced cell proliferation and unfavorable prognosis in glioma, lung adenocarcinoma, and colorectal cancer [16–18]. To investigate MCM4 expression in PCa, transcriptomic data from the public datasets GSE32269 and GSE77930 were analyzed. MCM4 mRNA levels were significantly higher in CRPC tissues compared to localized PCa samples (Fig. 6A, B). Similarly, analysis of the GSE70768 cohort revealed that patients with high GS (>7) exhibited significantly elevated MCM4 expression compared to those with lower GS (<7) (Fig. 6C). Furthermore, Kaplan–Meier survival analysis of The Cancer Genome Atlas (TCGA–PRAD) dataset demonstrated that increased MCM4 mRNA expression correlated with poorer overall survival (*p* = 0.00042), supporting a role for MCM4 in PCa progression (Fig. 6D).

To further explore the relationship between AR signaling and MCM4, MCM4 expression was assessed at both the mRNA and protein levels in CRPC cell lines (22Rv1 and C4-2B). Combined treatment with ENZ and DAL led to a more pronounced reduction in MCM4 expression compared to either agent alone (Fig. 6E, F). Additionally, previous studies have reported that MCM4 can activate the phosphatidylinositol 3ʹ-kinase (PI3K)/AKT signaling pathway [14, 19]. Thus, the effect of ENZ + DAL on this pathway was examined. Western blot analysis demonstrated that the combination treatment significantly suppressed PI3K/AKT signaling activity (Fig. 6F).

Given that AR functions as a transcription factor, it may directly regulate MCM4 transcription. To identify potential AR-binding sites, this study further analyzed the MCM4 promoter using the JASPAR database and predicted three putative androgen response elements (AREs), designated P1, P2, and P3 (Fig. 6G). Based on these predictions, this study cloned the MCM4 promoter into a dual-luciferase reporter construct and generated a series of promoter truncation and site-directed mutant constructs, including ΔP2/P3 (retaining only P1), ΔP3 (retaining P1 and P2), and mP3 (harboring a point mutation in the P3 site: 5ʹ-GGGAACTCTGGGTCT-3ʹ). Luciferase assays in 293 T cells showed that AR overexpression significantly enhanced MCM4 promoter activity, supporting AR-mediated transcriptional activation. Notably, deletion of the P2/P3 or P3 regions markedly reduced promoter activity and abolished AR-induced transactivation. Similarly, mutation of the P3 site (mP3) nearly eliminated AR-mediated activation, resembling the effect of ΔP3 deletion (Fig. 6H). These results identify the P3 site as the principal ARE mediating AR-dependent transcriptional regulation of MCM4, while the P1 and P2 sites appear dispensable in this context.

Considering that CDK4/6 inhibitors share similar mechanisms of action, this study further investigated whether the cooperative effect observed with DAL could extend to other agents in this class. CRPC cells were treated with palbociclib or ribociclib for 48 h, either alone or in combination with ENZ. Western blot analysis revealed that the protein levels of MCM4, p-RB, and E2F1 were significantly reduced in the combination treatment groups compared to monotherapy groups, indicating a consistent inhibitory effect of CDK4/6 inhibition in enhancing the therapeutic efficacy of ENZ (Supplementary Fig. S4A, B). These results suggest that the synergistic effect of combining ENZ with CDK4/6 inhibition extends beyond DAL and represents a broader class effect. These results indicate that AR inhibition synergizes with CDK4/6 blockade to repress MCM4 transcription, thereby enhancing the inhibitory effects on DNA replication and cell cycle progression in CRPC cells.

### AR and CDK4/6 mediate the synergistic antitumor effects of enzalutamide and dalpiciclib

To determine whether the antitumor effects of ENZ and DAL depend on AR and CDK4/6 activity, AR or CDK4/6 expression was silenced using siRNA in 22Rv1 and C4-2B cells. Efficient knockdown was confirmed by qPCR and immunoblotting (Supplementary Fig. S5A, B). The effects on key regulators involved in AR signaling, RB phosphorylation, and DNA replication were then examined.

AR knockdown significantly reduced the expression of MCM4, phosphorylated RB (p-RB), and E2F1, consistent with the suppression of AR-dependent transcriptional and cell cycle signaling. ENZ treatment produced a similar inhibitory effect in control cells. In AR-silenced cells, ENZ did not induce further changes in these downstream targets, suggesting that the biological effects of ENZ are primarily mediated through AR inhibition (Fig. 6I, Supplementary Fig. S6A).

Similarly, CDK4/6 depletion resulted in a marked reduction in RB phosphorylation and E2F1 expression, mimicking the molecular effects observed with DAL treatment. In CDK4/6-silenced cells, DAL caused minimal additional suppression of p-RB or E2F1, indicating that DAL exerts its effects predominantly through CDK4/6-dependent RB pathway blockade. Importantly, combined silencing of AR or CDK4/6 with pharmacological inhibition further suppressed MCM4 expression and RB-E2F signaling, consistent with cooperative inhibition of DNA replication and cell cycle progression (Fig. 6J, Supplementary Fig. S6B).

These results demonstrate that ENZ and DAL exert their antitumor effects mainly through AR- and CDK4/6-dependent mechanisms, respectively, and converge on the RB-E2F-MCM4 axis to produce synergistic suppression of CRPC cell proliferation.

### The combination of enzalutamide and dalpiciclib exerts potent antitumor activity in vivo

**Fig. 7:**
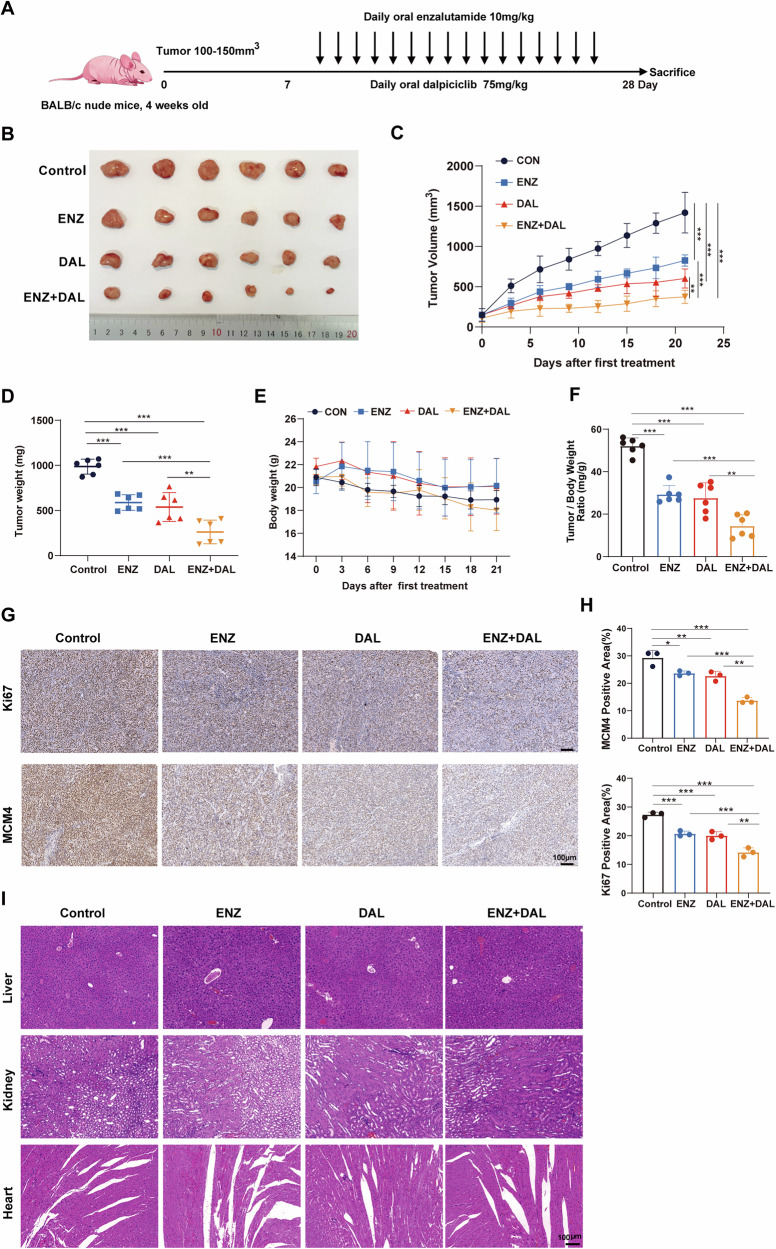
Synergistic suppression of CRPC tumor growth by ENZ and DAL in vivo. **A** Schematic illustrating the experimental design for evaluating the in vivo antitumor activity of ENZ and DAL in 22Rv1 and C4-2B xenograft models. **B** Representative images of 22Rv1 xenograft tumors harvested from each group at the experimental endpoint (day 28). **C** Tumor growth curves, presented as mean ± SD. **D** Tumor weights at the endpoint, presented as mean ± SD. **E** Body weight curves of mice during the treatment period. **F** Normalized tumor weight ratio. Tumor weights were normalized to the terminal body weight of mice in each group (*n* = 6). **G**, **H** Representative IHC staining images of Ki-67 (proliferation marker) and MCM4 in 22Rv1 xenograft tumors from each treatment group (scale bar = 100 μm). Right panels show quantification of Ki-67 and MCM4 expression levels. **I** Representative H&E staining images of vital organs (heart, liver, kidney) from mice bearing 22Rv1 tumors in the corresponding groups (scale bar = 100 μm). Data are presented as mean ± SD. Statistical significance was determined by one-way ANOVA with Tukey’s multiple comparisons test (**C**, **D**, **E**, **F**, **H**). *p < 0.05, **p < 0.01, ***p < 0.001.

To evaluate the in vivo antitumor efficacy of the ENZ + DAL combination, cell-derived xenograft (CDX) models were established using 22Rv1 and C4-2B CRPC cell lines (Fig. 7A). Combined administration of ENZ and DAL markedly inhibited tumor growth compared to either monotherapy in both models (Fig. 7B, C, Supplementary Fig. S7A, B). Tumors harvested from the combination-treated mice had significantly lower weights than those from the monotherapy groups (Fig. 7D, Supplementary Fig. S7C). Throughout the treatment, all mice tolerated the regimens well. Although a slight decrease in body weight was observed in the combination group, the difference was not statistically significant (Fig. 7E, Supplementary Fig. S7D). Furthermore, when tumor weight was normalized to terminal body weight, the combination group still exhibited a significantly lower tumor-to-body weight ratio compared to the monotherapy groups (Fig. 7F, Supplementary Fig. S7E), confirming that the antitumor effect was not a secondary result of systemic weight loss. These results demonstrate that the ENZ + DAL combination exerts robust antitumor activity in vivo without inducing notable systemic toxicity.

IHC analysis of xenograft tumors derived from 22Rv1 cells showed that expression of the proliferation marker Ki-67 and the replication-associated protein MCM4 was significantly reduced in the combination treatment group (Fig. 7G, H). Moreover, hematoxylin and eosin (H&E) staining of major organs from the 22Rv1 xenograft model, including the heart, liver, and kidneys, revealed no apparent histopathological abnormalities (Fig. 7I), indicating that the regimen is safe and well-tolerated.

## Discussion

PCa remains the second leading cause of cancer-related mortality among men in Western countries [20]. ADT is the standard of care for patients with recurrent or metastatic disease following curative local treatment [21–23]. However, ADT provides only transient disease control, as most patients eventually progress to CRPC, a lethal stage of the disease with limited therapeutic options. Previous studies have established a strong biological rationale for targeting the Cyclin D-CDK4/6-RB axis in PCa, where dysregulation of cell-cycle control is common and closely associated with AR signaling. Multiple clinical trials have been initiated to evaluate CDK4/6 inhibitors in various disease settings. However, the molecular mechanisms underlying the interaction between CDK4/6 blockade and AR inhibition remain incompletely defined.

This study demonstrates a pronounced synergistic antitumor effect between the AR inhibitor ENZ and the CDK4/6 inhibitor DAL in both in vitro and in vivo CRPC models. Combined treatment with ENZ and DAL significantly inhibited cell proliferation, induced G1-phase cell cycle arrest, suppressed migratory and invasive capacities, and promoted apoptosis in 22Rv1 and C4-2B cells. In vivo, the ENZ + DAL combination produced superior tumor growth inhibition compared to either monotherapy, without notable systemic toxicity. These findings provide robust preclinical evidence supporting the combination of AR and CDK4/6 targeting as a promising therapeutic approach for CRPC. Notably, preclinical models typically assess short-term therapeutic efficacy, and the effects of long-term treatment remain to be explored in future clinical studies.

**Fig. 8:**
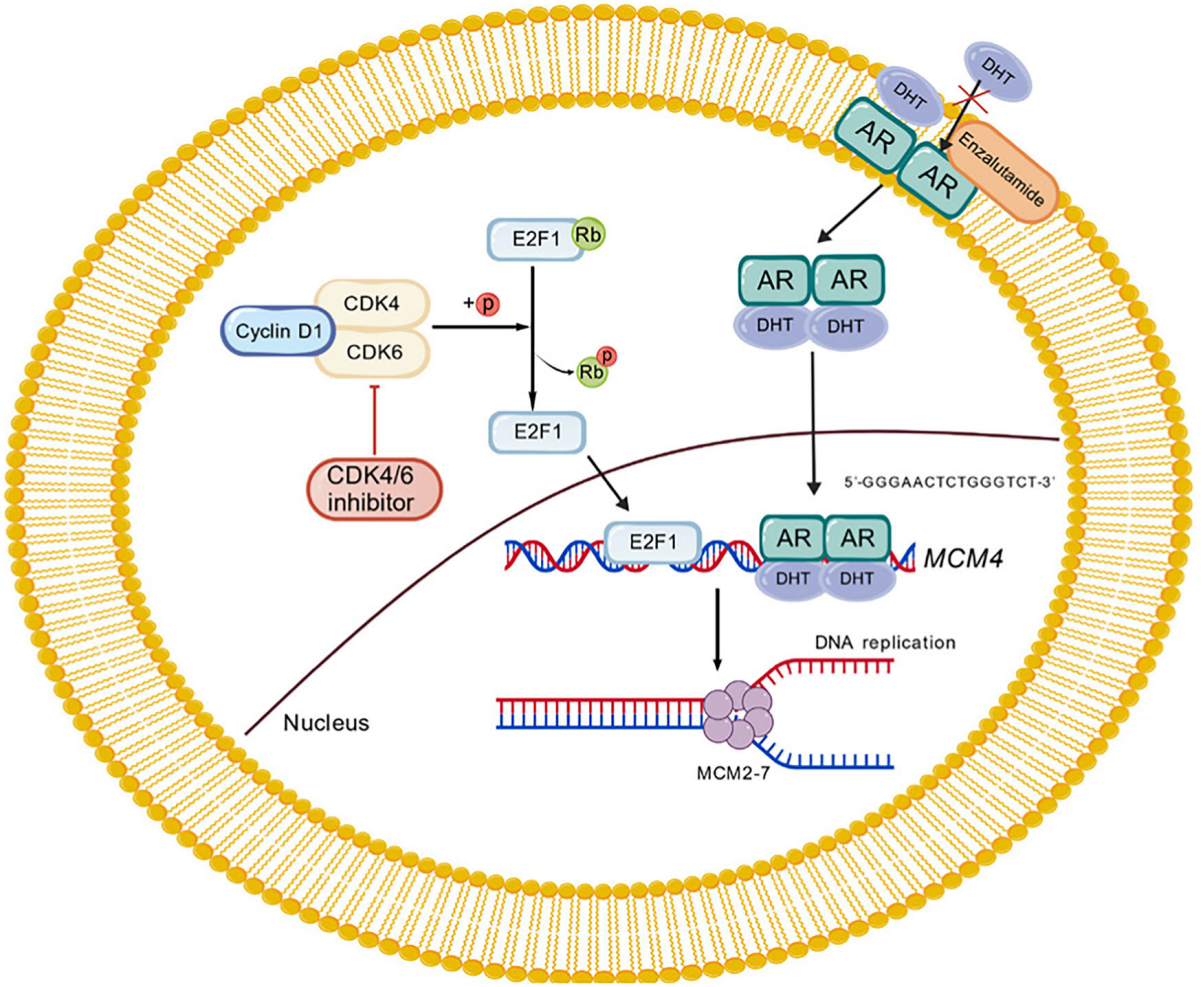
Mechanistic model of the study. Dihydrotestosterone (DHT) binds to the androgen receptor (AR). The activated AR translocates to the nucleus and binds to specific DNA sequences (5’-GGGAACTCTGGGTCT-3’), regulating MCM4 gene expression. This action indirectly enhances MCM4 transcription via the key transcription factor E2F1, promoting the formation of the DNA replicative helicase complex MCM2-7 and facilitating DNA replication initiation. Enzalutamide (ENZ), by inhibiting AR-mediated MCM4 expression, synergistically enhances the suppression of the cell cycle and DNA replication induced by the CDK4/6 inhibitor.

Mechanistically, our data reveal a novel axis underlying the synergy between ENZ and DAL. While DAL primarily induces G1-phase arrest through CDK4/6 inhibition, ENZ enhances this effect and further augments DNA replication suppression. Transcriptomic analysis identified significant downregulation of DNA replication-associated pathways, particularly involving MCM4, a core subunit of the MCM helicase complex responsible for DNA unwinding during replication. Luciferase reporter assays confirmed that AR directly regulates MCM4 transcription, and AR inhibition by ENZ represses MCM4 expression, thereby potentiating DAL-induced blockade of DNA replication. These results establish a mechanistic link between AR signaling and replication control, with MCM4 acting as a key mediator of ENZ-DAL synergy in CRPC (Fig. 8).

In summary, the combination of ENZ and DAL exhibits synthetic lethality in CRPC by concurrently disrupting AR-driven transcription and CDK4/6-mediated cell cycle regulation. This dual inhibition impedes DNA replication and induces tumor regression in preclinical CRPC models. Our findings provide comprehensive mechanistic insight and a strong experimental rationale for further clinical evaluation of the ENZ-DAL combination as a novel therapeutic strategy for advanced PCa.

## Conclusions

CDK4/6 inhibition has emerged as a promising targeted therapeutic approach across various cancer types. In PCa, frequent dysregulation of the Cyclin D-CDK4/6-RB axis, coupled with strong preclinical evidence, highlights its therapeutic potential. This study demonstrates a robust synergistic interaction between the AR inhibitor ENZ and the CDK4/6 inhibitor DAL in CRPC models, as confirmed by the Chou-Talalay method. The synergy was consistently validated through comprehensive assays and in vivo CDX models using both 22Rv1 and C4-2B cells. Notably, combination treatment significantly reduced LN metastasis in a popliteal LN metastasis model established with GFP-labeled 22Rv1 cells, further supporting the in vivo antitumor efficacy of this strategy.

Mechanistically, combined AR blockade and CDK4/6 inhibition cooperatively suppressed MCM4-mediated DNA replication, enhancing antitumor efficacy. These findings provide compelling experimental and mechanistic evidence supporting the ENZ-DAL combination as a rational and potentially translatable therapeutic strategy for CRPC.

## Materials and methods

### Cell lines and reagents

The human prostate cancer cell lines 22Rv1 and C4-2B were kindly provided by the Cell Bank of the Chinese Academy of Sciences. All cells were maintained in RPMI 1640 complete medium (Hyclone, USA), supplemented with 10% fetal bovine serum (FBS; Gibco, USA), 100 U/mL penicillin (Biosharp, China), and 100 μg/mL streptomycin (Biosharp, China). Cells were cultured at 37 °C in a humidified atmosphere containing 5% CO₂. All cell lines were routinely authenticated and tested for mycoplasma contamination. GFP-22Rv1 cells were generated by lentiviral transduction using a GFP-expressing vector. Briefly, 22Rv1 cells were infected with lentiviral particles encoding green fluorescent protein (GFP) in the presence of polybrene (8 μg/mL). After 48 h, transduced cells were selected with puromycin (2 μg/mL) for 7–10 days to establish stable GFP-expressing cells. GFP expression was confirmed by fluorescence microscopy prior to subsequent experiments. For cell-based experiments, samples were allocated to experimental groups without formal randomization.

Enzalutamide (Selleck, USA) and dalpiciclib (MedChemExpress, USA) were dissolved in dimethyl sulfoxide (DMSO; Sigma, USA) and stored at −20 °C.

### Cell viability assay

22Rv1 and C4-2B cells were cultivated in RPMI 1640 complete medium. Approximately 6000 cells per well were seeded in 96-well plates. After overnight incubation, cells were exposed to a 2-fold serial dilution of ENZ, ranging from 10 to 160 μM, and DAL, ranging from 1 to 16 μM for 48 h. Cell viability was assessed using Cell Counting Kit 8 (CCK-8, Beyotime, China) following the manufacturer’s protocol. Cell viability was determined by the percentage of surviving drug-treated cells versus DMSO-treated control cells. The IC_50_ value was defined as the concentration required to achieve 50% inhibition of cell proliferation. For combination treatment experiments, cells were treated with the indicated concentrations of ENZ and/or DAL (10:1 ratio) for 48 h.

### Drug synergy analysis

Drug combination synergy analysis was performed using the Chou-Talalay method[24]. The combination index (CI) values were calculated using CompuSyn software to quantify the interaction between the two drugs. CI values at different fraction affected (Fa) levels were generated using CompuSyn software. According to the CI theorem, values of CI < 1, CI = 1, and CI>1 indicate synergism, an additive effect, and antagonism, respectively.

### Colony formation assay

For the colony formation assay, 1,000 cells were seeded into each well of a 6-well plate. After overnight incubation, the cells were treated with DMSO, ENZ (35 μM), DAL (2 μM), ENZ (35 μM) + DAL (2 μM) for 12 to 17 days, with the medium replaced every three days. Following treatment, the colonies were fixed with formaldehyde and stained with 0.5% crystal violet. Only colonies consisting of 50 or more cells were counted and subjected to statistical analysis.

### 5-Bromo-2’-deoxyuridine (BrdU) Cell Proliferation Assay

22Rv1 and C4-2B cells were seeded in 6-well plates at a density of 4×10⁶ cells per well. After 48 h of drug treatment, the cells were incubated with 10 μM BrdU (Sigma, B5002, USA) for 6 h, followed by washing with PBS and fixation with 4% paraformaldehyde. HCl working solution was then added and incubated at 37 °C for 40 minutes. After another fixation step with 4% paraformaldehyde, cells were blocked with 3% bovine serum albumin (BSA). The cells were incubated overnight at 4 °C with an anti-BrdU antibody (Millipore, 05-633, USA, 1:200) diluted in 1% BSA, washed three times (5 minutes each) with PBS, and then incubated with an appropriate secondary antibody (Beyotime, A0521, 1:300, China). DNA was stained using Gold Antifade Mountant with DAPI (Invitrogen, USA, P36931). Images were acquired using a 3DHISTECH Pannoramic MIDI scanner.

### 3D spheroid formation assay

Treated cells were resuspended in preformulated spheroid formation medium. The cells were seeded uniformly at a density of 3000 cells per well in ultra-low attachment 96-well plates and cultured for 2–3 weeks at 37 °C under 5% CO₂. Plate movement was strictly avoided during the first three days. The medium was replenished periodically throughout the culture period. Spheroids were observed and imaged under a microscope. A spheroid consisting of 50 or more cells was defined as a positive spheroid and counted for subsequent analysis. To evaluate the inhibitory effects of treatments, the maximum diameter of each spheroid was measured using ImageJ software. For each group, the average maximum diameter was calculated across three independent biological replicates (*n* = 3). The spheroid formation medium comprised the following components: B27 Supplement (Gibco, 17504044, USA); N-27 Supplement (Gibco, 17502001, USA); HumanKine® recombinant human EGF protein (Proteintech, HZ-1326, China); HumanKine® recombinant human FGF-8b protein, GMP grade (Proteintech, HZ-1103_GMP, China); DMEM/F12 basal medium (Gibco, 12634010, USA).

### Flow cytometric analysis

Flow cytometry was performed to assess cell apoptosis and cell cycle distribution. 22Rv1 and C4-2B cells were seeded and treated with DMSO, ENZ (35 μM), DAL (2 μM), or the combination of ENZ (35 μM) + DAL (2 μM). After 48 h, cells were digested using EDTA-free trypsin. For apoptosis evaluation, cells were collected and stained with an Annexin V-FITC/PI Apoptosis Kit (Liankebio, China, AT101). For cell cycle analysis, cells were fixed overnight in 70% precooled ethanol at 4 °C, washed with PBS, and stained with a PI solution (MultiSciences, China, AP101). All stained cells were analyzed and quantified according to the manufacturer’s instructions using a flow cytometer (CytoFLEX, Beckman, USA). Data were processed with FlowJo v10 software.

### Western blotting analysis

Cells were treated with DMSO, ENZ (35 μM), DAL (2 μM), or the combination of ENZ (35 μM) + DAL (2 μM) for 48 h. Cells were harvested and lysed in RIPA buffer (Beyotime, China) in the presence of protease and phosphatase inhibitors (Roche, USA). Protein concentration was quantified using a BCA assay (Thermo Fisher Scientific, Italy). The lysates were mixed with 1× loading buffer (Beyotime, China). A total of 30 μg protein per sample was loaded onto 7.5% or 10% SDS-PAGE gels, which were run for 1 hour at 150 V in the running buffer (25 mM Tris base, 192 mM glycine, 0.1% SDS). Proteins were then transferred from the gels onto activated PVDF membranes (Tanon, Shanghai, China) at 100 V for 90 minutes in the transfer buffer (25 mM Tris base,192 mM glycine, 10% methanol). Membranes were blocked with 5% BSA for 1 h. Subsequently, the membranes were incubated overnight at 4 °C with primary antibodies. Membranes were incubated with HRP-conjugated goat anti-mouse or goat anti-rabbit secondary antibodies for 60 minutes at room temperature. The immunoreactive proteins were detected using enhanced chemiluminescence (P0018S, Beyotime, China). Information on the antibodies used in this study is provided in Supplementary Table S1.

### Wound healing assays

For wound healing assays, cells were manually scratched once they reached near-confluence in 6-well plates. After scratching, the cells were cultured in serum-free medium to minimize the effect of cell proliferation on wound closure. Images of the wounded area were captured immediately after scratching (0 h) and again after 24 h of incubation. All resulting images were analyzed using ImageJ software (version 1.53).

### Migration assay

For the Transwell migration assay (Corning, USA), cells were treated with DMSO, ENZ (35 μM), DAL (2 μM), or the combination of ENZ (35 μM) + DAL (2 μM) for 48 h. Subsequently, 5–10 × 10^4^ cells were resuspended in 100 μL of serum-free medium and seeded into the upper chamber of each insert, with the lower chamber filled with 600 μL of medium supplemented with 20% fetal bovine serum. The Transwell plate was incubated at 37 °C in a humidified atmosphere with 95% air and 5% CO_2_ for 48 h. After incubation, the chambers were rinsed with PBS, fixed with formaldehyde, and stained with 0.5% crystal violet. Migrated cells were imaged and counted using ImageJ software (National Institutes of Health, Bethesda, MD, USA).

### RNA-Seq analysis

Total RNA was extracted from 22Rv1 cells treated with DMSO, ENZ (35 μM), DAL (2 μM), or ENZ (35 μM) + DAL (2 μM) for 48 h using TRIzol Reagent (Invitrogen). RNA quantity and quality were assessed by an Agilent 2100 Bioanalyzer (Agilent Technologies, Palo Alto, CA, USA), NanoDrop spectrophotometer (Thermo Fisher Scientific Inc.), and 1% agarose gel electrophoresis. Samples with a RIN > 6.5 were used for library preparation. Sequencing libraries were prepared using the Hieff NGS® Ultima Dual-mode mRNA Library Prep Kit (Yeasen, 12309ES) and sequenced on a DNBSEQ-T7 platform. Raw reads were filtered using fastp (v0.18.0), and rRNA reads were removed using Bowtie2 (v2.2.8). Clean reads were aligned to the human reference genome (GRCh38) using HISAT2 (v2.1.0). Transcript assembly and expression quantification were performed using StringTie (v1.3.1) and RSEM, respectively. GSEA was performed using the Java GSEA program with the MSigDB Hallmark gene set collection.

### RNA Isolation and quantitative reverse transcription PCR (qRT-PCR)

Total RNA from cells was extracted using the Total RNA Kit II (R6934-01, Omega, Georgia, USA). RNA samples were quantified with Nanodrop. cDNAs were synthesized using the Reverse Transcription Kit (R223-D1, Vazyme Biotechnology, China). Gene expression was quantified using ChamQ SYBR qPCR Master Mix (Q311-02, Vazyme Biotechnology, China) via the delta-delta CT method, with β-actin used as the housekeeping gene. The primers used to amplify the indicated genes are listed in the Supplementary Material (Supplementary Table S2).

### Dual-luciferase reporter assay

293 T cells were cultured in 24-well plates until approximately 60% confluence. Plasmids with the desired combinations were then transfected into the cells and incubated for 48 h. Putative AR-binding sites in the MCM4 promoter were predicted using the JASPAR database (JASPAR: a database of transcription factor binding profiles; https://jaspar.elixir.no/). Specific experimental details adhered to the protocols outlined in the Dual-Luciferase Reporter Assay System kit (E1910, Promega).

### siRNA transfection

Small interfering RNAs (siRNAs) targeting CDK4, CDK6, and AR were purchased from GenePharma (Shanghai, China), with corresponding sequences provided in Supplementary Table S3. Transient transfection of siRNAs or plasmids was performed using Lipofectamine™ 3000 Transfection Reagent (L3000075, Invitrogen, USA) according to the manufacturer’s instructions. Cells were harvested at specified time points for subsequent experiments.

### Animal experiments

All animal experiments were approved by the Experimental Animal Welfare Ethics Committee of Zhongnan Hospital of Wuhan University (approval number: ZN2024217) and were conducted following the guidelines of the Institutional Animal Care and Use Committee and the National Institutes of Health (NIH) Guide for the Care and Use of Laboratory Animals. Male BALB/c nude mice (4 weeks old) were purchased from Hubei Bennt Biotechnology Co., Ltd. (Wuhan, China).

To evaluate the therapeutic efficacy of ENZ in combination with DAL, CDX models were established using 22Rv1 and C4-2B PCa cells. Briefly, 3 × 10^6^ cells suspended in 100 μL phosphate-buffered saline (PBS) were subcutaneously injected into the flanks of nude mice. When tumors reached approximately 100 mm^3^, the mice were randomly assigned to one of four groups (n = 6 per group): control, ENZ, DAL, and combination treatment (ENZ + DAL). No blinding was performed for the treatment groups. Mice in the control group received saline by oral gavage. The ENZ group was treated with ENZ (10 mg/kg, oral gavage, once daily for 21 days), the DAL group received DAL (75 mg/kg), and the combination group received both ENZ (10 mg/kg) and DAL (75 mg/kg). Tumor size and body weight were measured every three days. Tumor volume (V) was calculated using the formula: V = π/6 × length × width^2^.

In the popliteal LN metastasis model, 5 × 10^5^ GFP-22Rv1 cells suspended in 50 μL PBS were injected into the left hind footpad of nude mice (5 weeks old). Seven days after tumor cell injection, mice were randomly divided into four groups (n = 5 per group) and treated with the same regimens as described for the CDX model, including saline (control), ENZ (10 mg/kg), DAL (75 mg/kg), or the combination of ENZ and DAL for 21 days. No blinding was performed for the treatment groups. At the end of the treatment period, mice were sacrificed, and the popliteal LNs were harvested. GFP fluorescence signals in the popliteal LNs were detected using an IVIS in vivo imaging system (PerkinElmer, USA). The LNs were subsequently excised, and their size and weight were measured for further analysis.

### Immunohistochemistry (IHC)

IHC analysis was conducted on xenograft tumor specimens. Formalin-fixed, paraffin-embedded tissue sections (4 μm thick) were deparaffinized and rehydrated through a series of graded ethanol solutions (100%, 95%, 80%, 70%). After antigen retrieval, tissue slides were incubated overnight at 4 °C with primary antibodies against Ki-67 (1:200, #9027S, Cell Signaling Technology, USA), MCM4 (1:200, #9027S, Cell Signaling Technology, USA), and GFP (1:200, #ab183734, Abcam, USA). Following PBS washes, the slides were incubated with a horseradish peroxidase (HRP)-conjugated secondary antibody for 30 mins, and stained with diaminobenzidine (DAB) for 5 mins.

For H&E staining, tissue samples from the heart, liver, and kidneys were embedded, sectioned, deparaffinized, and rehydrated. The sections were then stained with hematoxylin for 3 minutes, followed by eosin for 30 secs.

### Public datasets

Genomic and transcriptomic data for TCGA–PRAD were retrieved from cBioPortal (https://www.cbioportal.org/) and analyzed using R software. Public gene expression datasets were obtained from the Gene Expression Omnibus (GEO) database for subsequent bioinformatics analysis. Pan-cancer genomic dependency data were sourced from the DepMap portal (DepMap Public 25Q2 dataset, https://depmap.org/portal/).

### Statistical Analysis

Survival analysis was performed using R software (version 4.4.2), and other statistical analyses were conducted with GraphPad Prism software (version 9.5). Data are presented as the mean ± standard deviation (SD) from at least three independent experiments, unless stated otherwise. Data normality was assumed but not formally tested. Survival analysis was conducted using the log-rank test. Statistical comparisons between two groups were performed using a two-tailed unpaired Student’s t-test, while one-way ANOVA with Tukey’s multiple comparisons test was used for comparisons between more than two groups. Survival differences were analyzed using the log-rank test. Statistical significance is indicated as follows: *p < 0.05, **p < 0.01, ***p < 0.001. The schematic diagram was created using BioGDP (https://biogdp.com/) [25].

## Supplementary information

Supplementary Figures S1–S7

Supplementary Tables S1–S3

uncropped western blot

## Data Availability

The RNA-seq data in the research have been uploaded to the GEO database with the accession code (GSE325471). Additional data are provided in the article or Supplementary Information. The uncropped Western blot data were provided in the Supplementary Material. Figure 8 was created with BioGDP software.
