# Supplementary material for: The CDK4/6 inhibitor dalpiciclib augments the antitumor efficacy of enzalutamide in preclinical models of castration-resistant prostate cancer through inhibition of MCM4-mediated DNA replication
Source: Cell Death Dis. 2026 Jun 5;17(1):703. doi: 10.1038/s41419-026-08959-9 (PMC13454615; doi:10.1038/s41419-026-08959-9)

Fig3

E

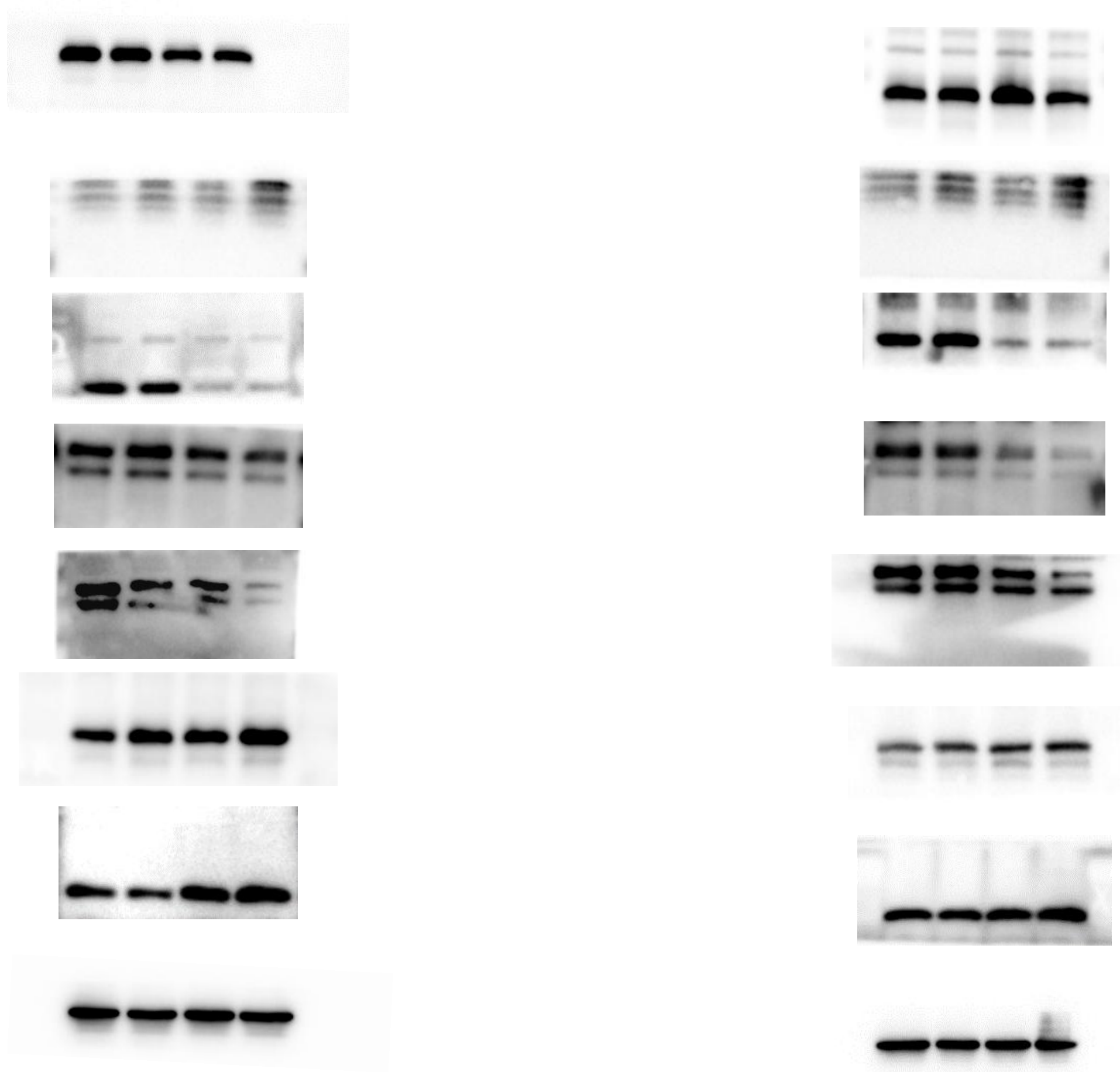

F

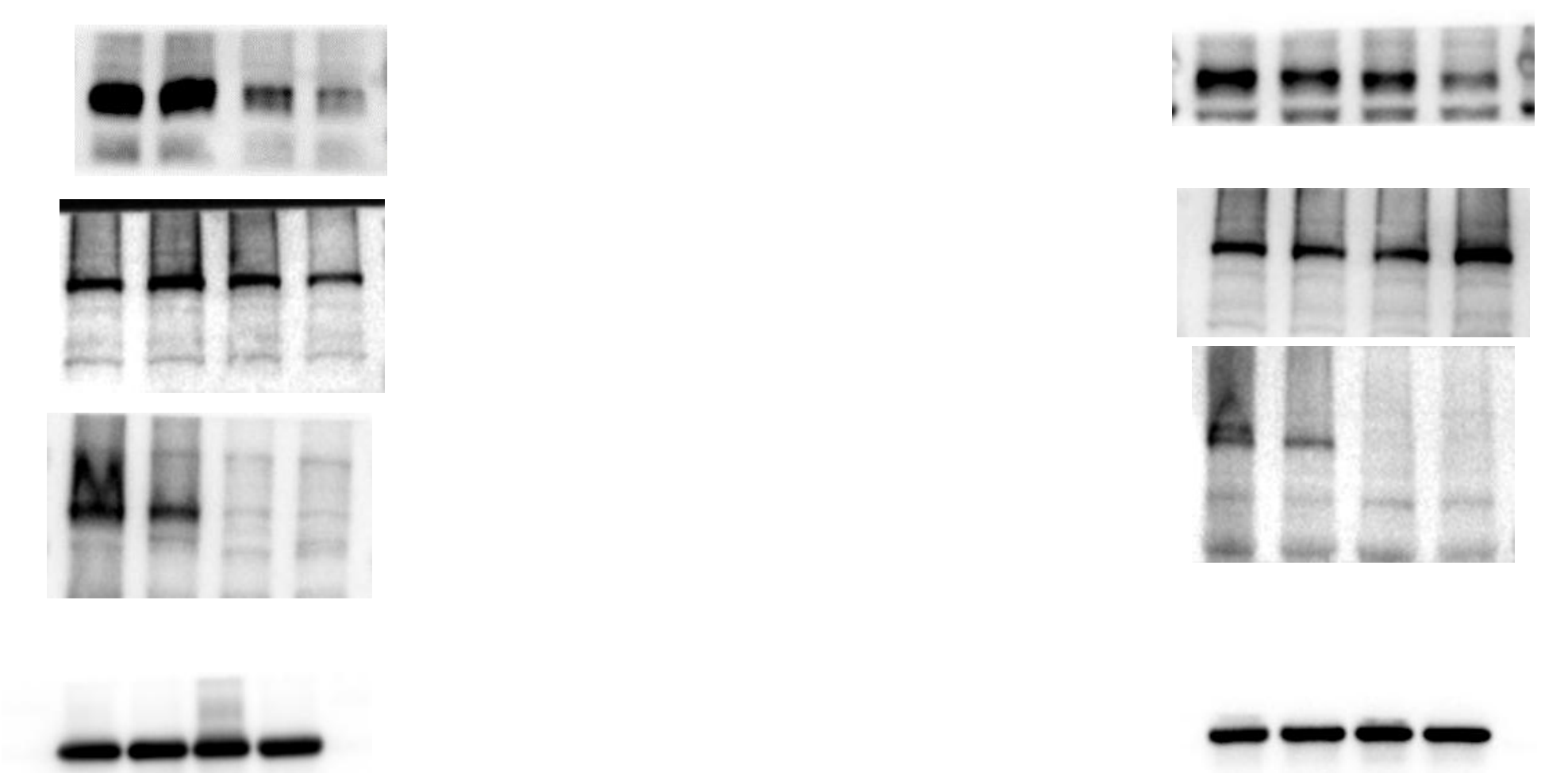

Fig4

F

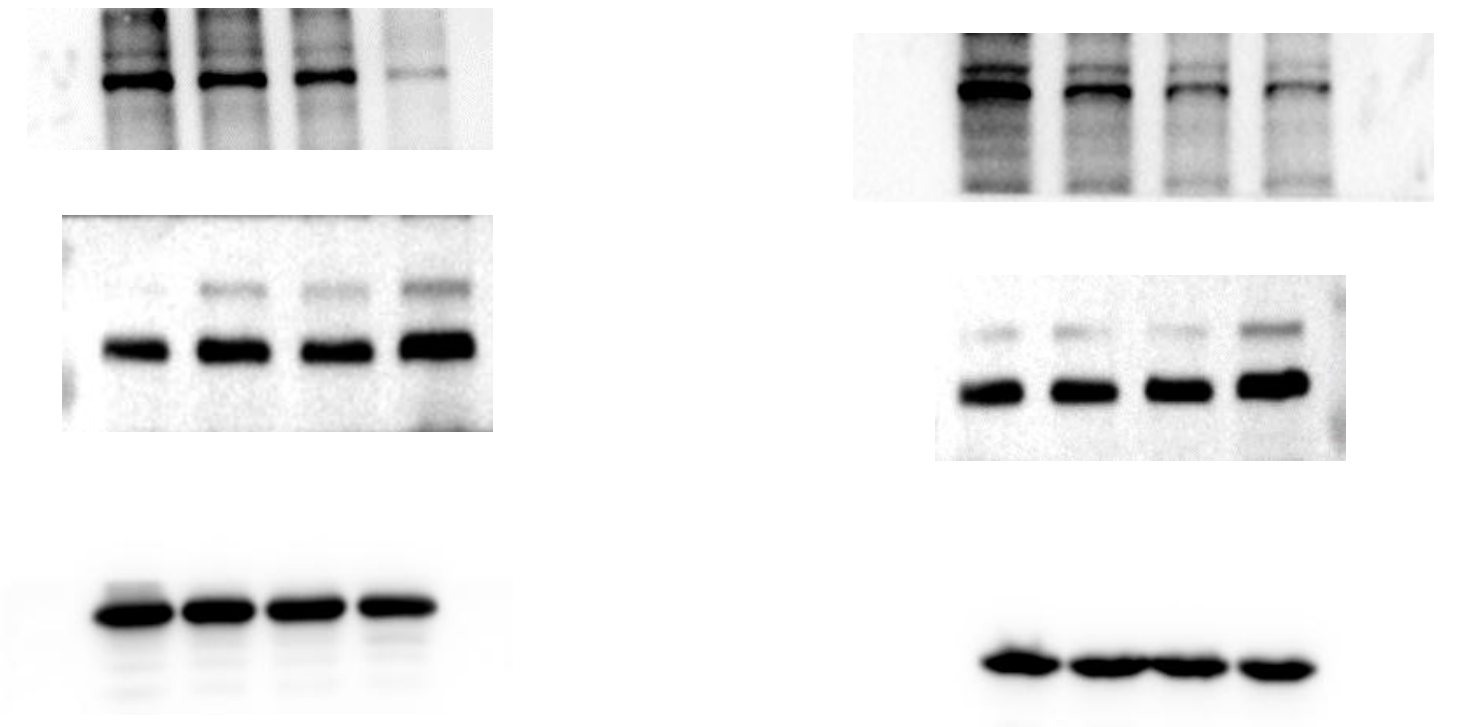

Fig6

F

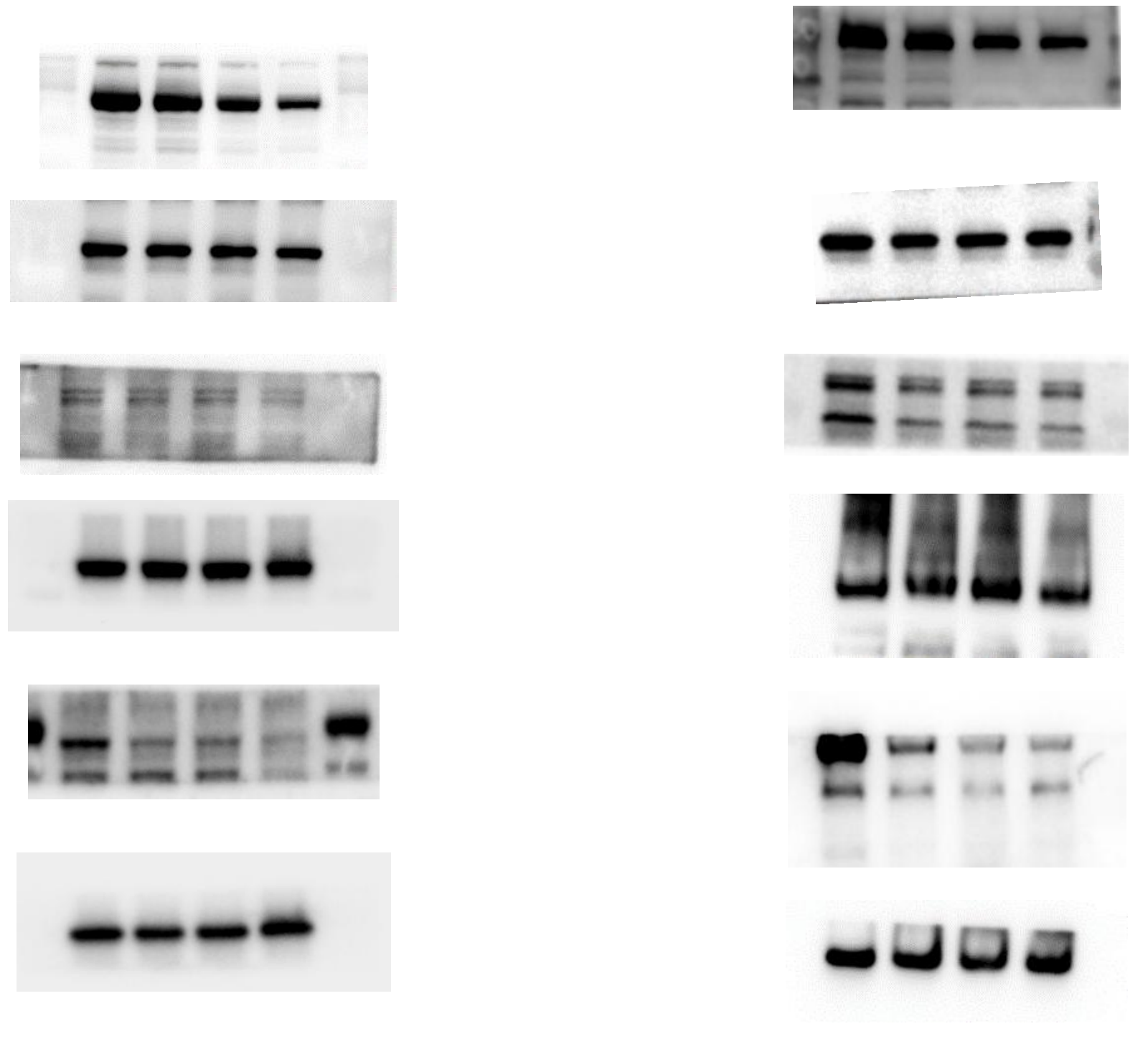

I

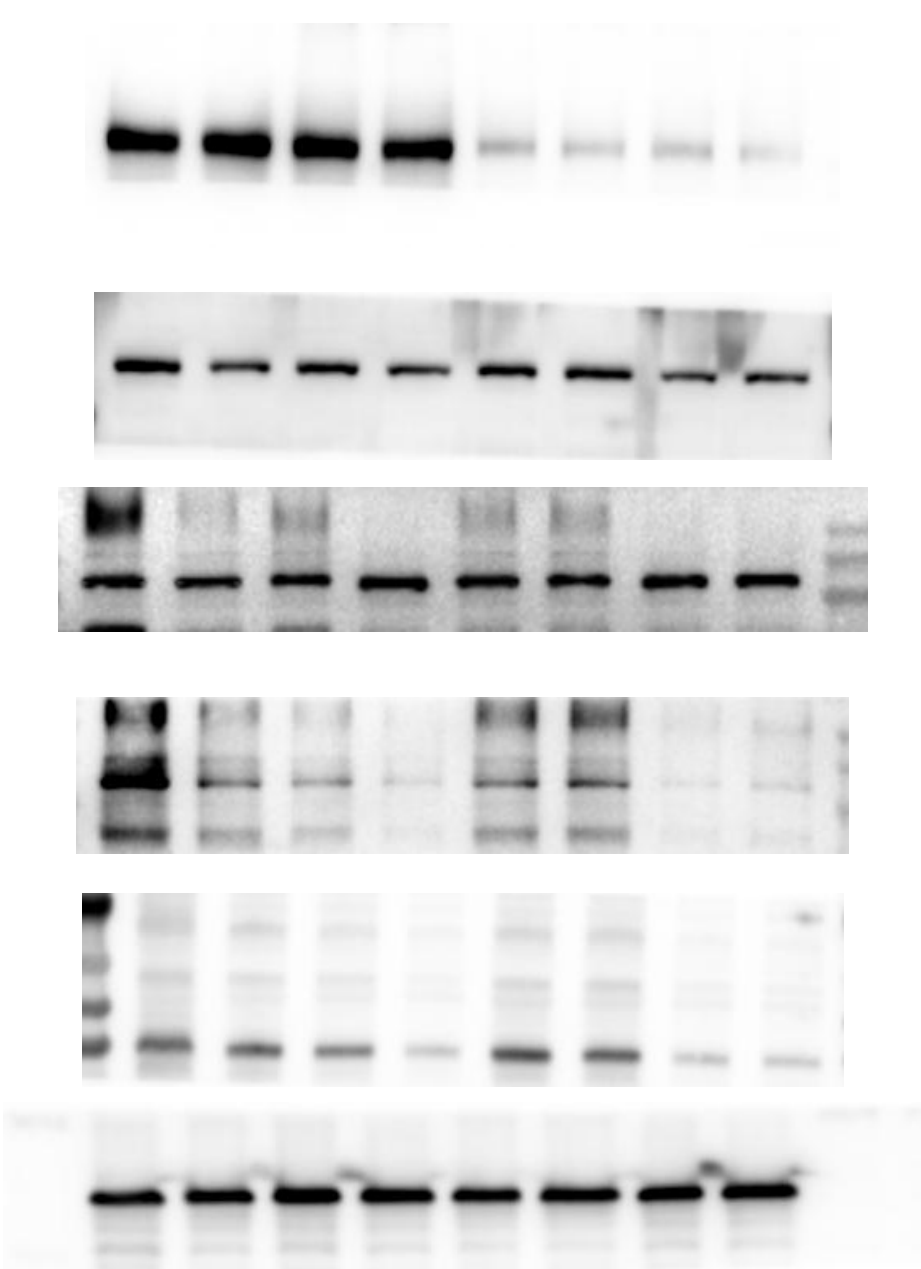

J

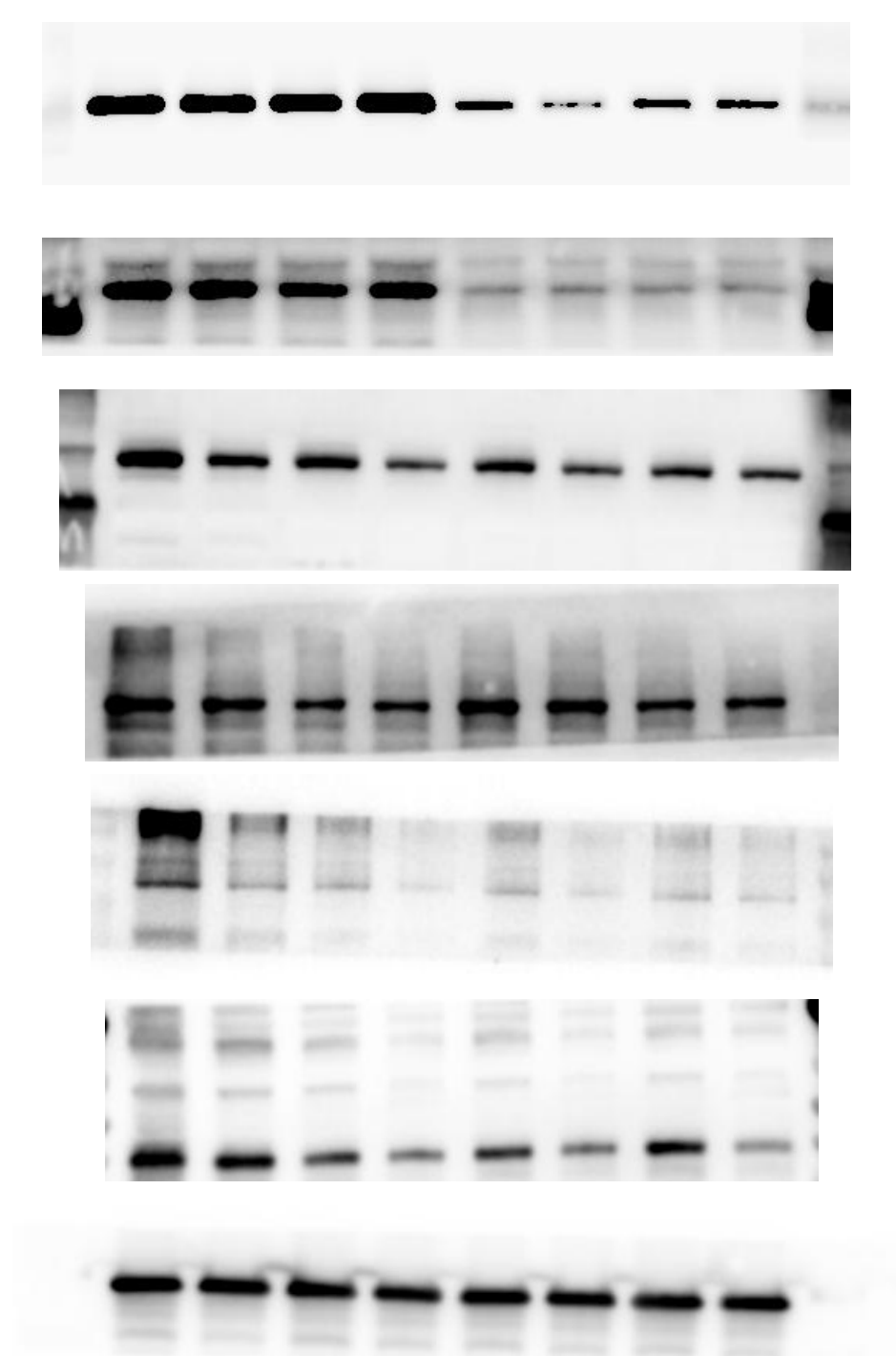

FigS4

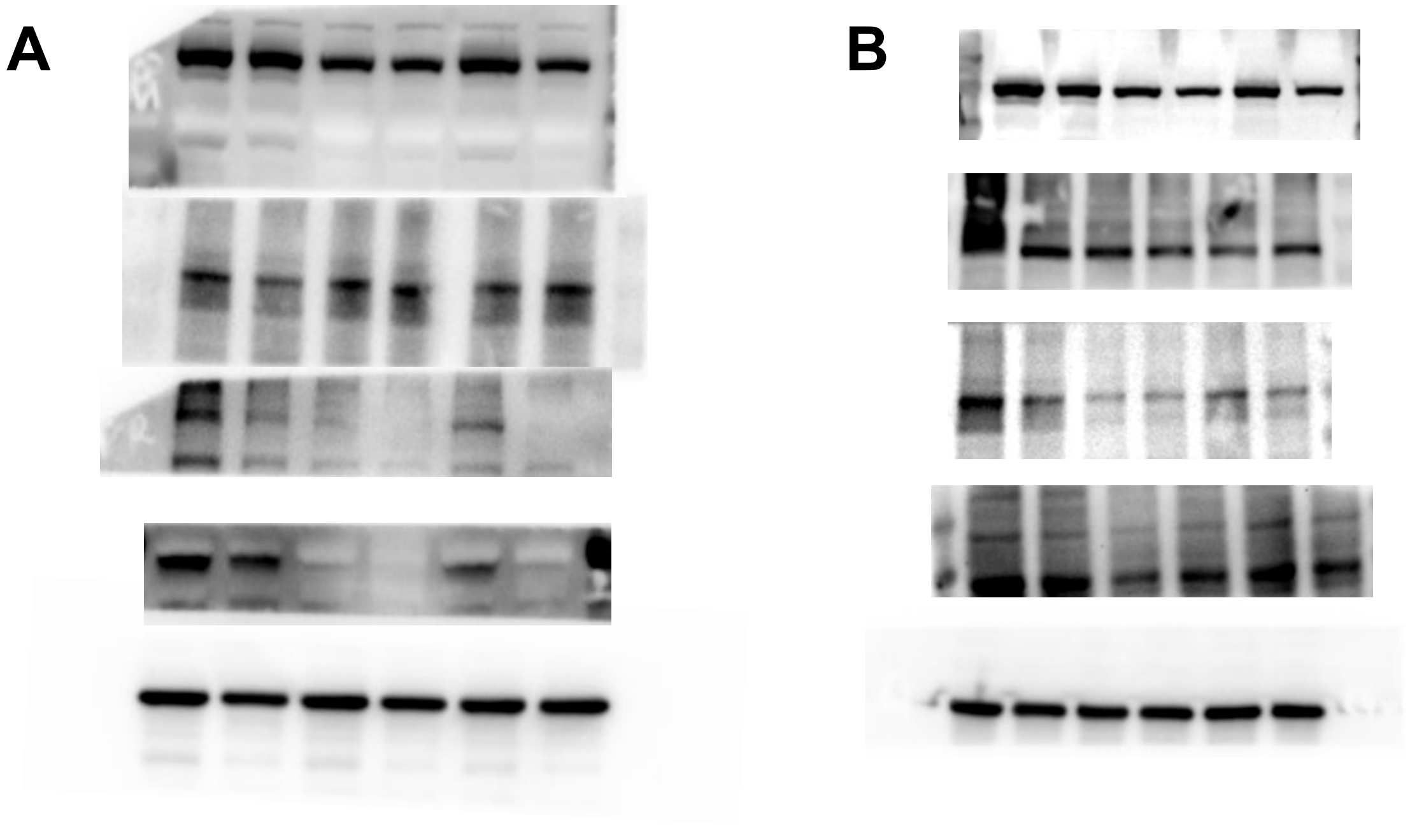

FigS5

B

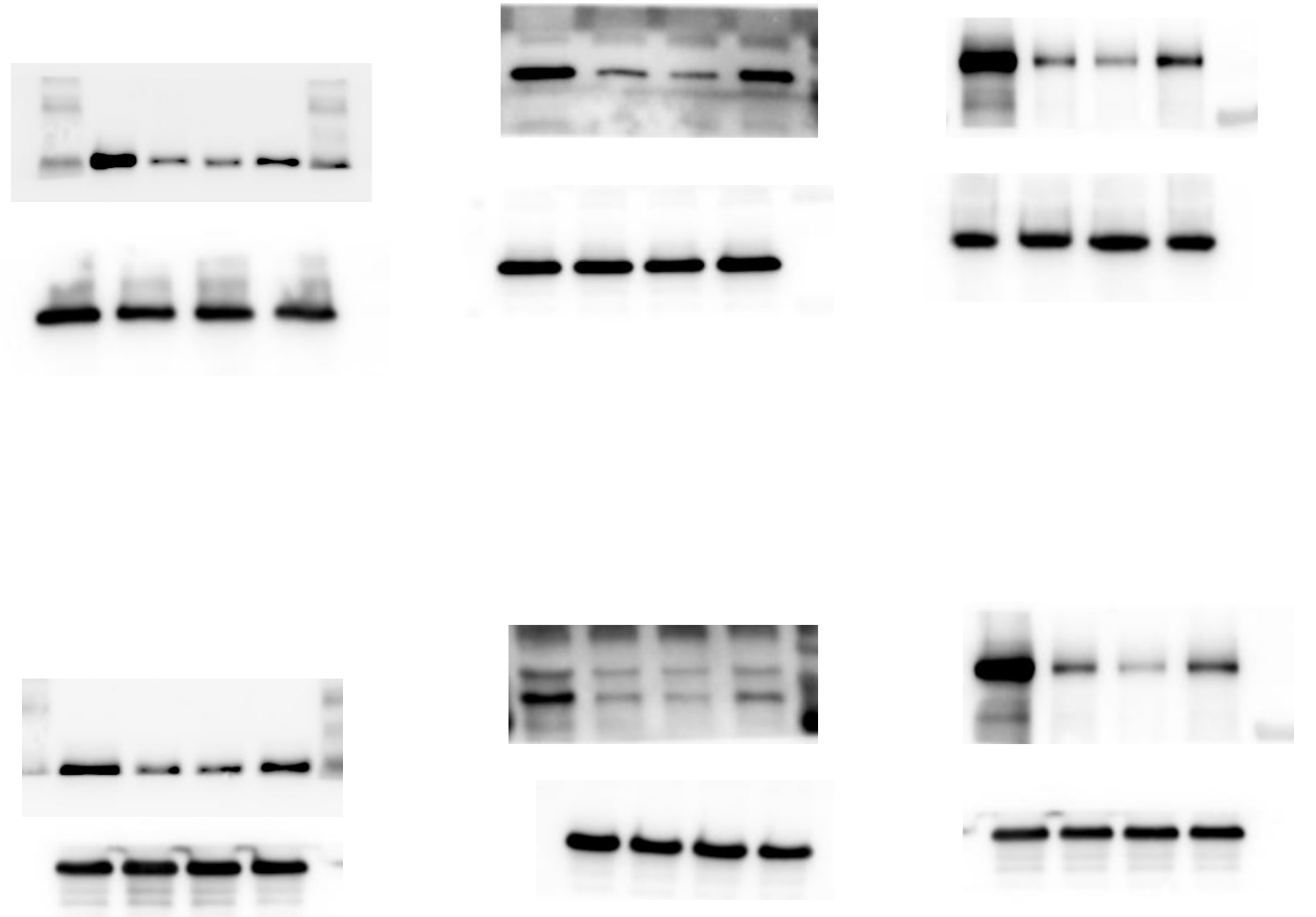

FigS6

A

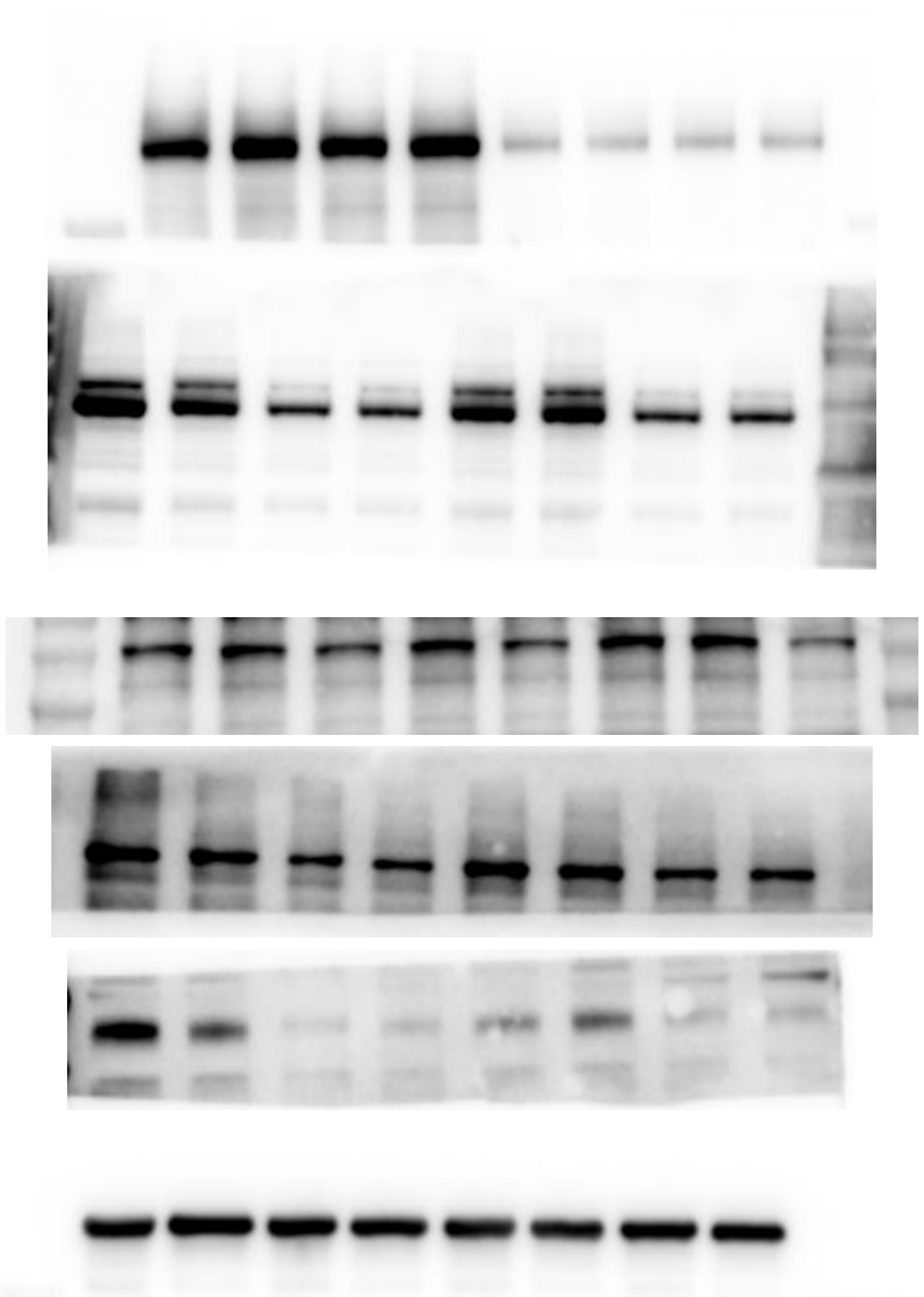

B

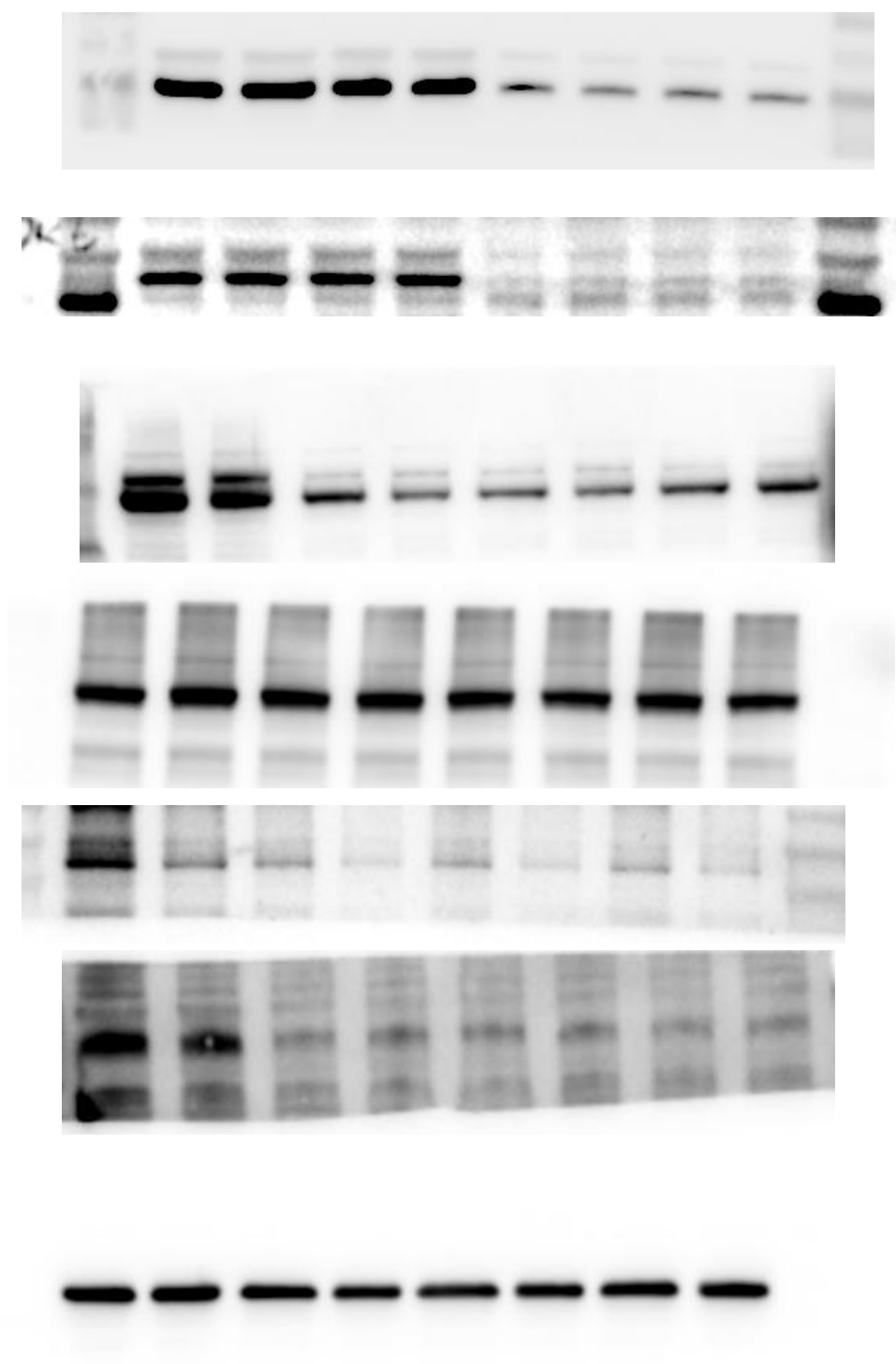

Supplement: Supplementary file 1 [file 41419_2026_8959_MOESM3_ESM.pdf]
