# Supplementary material for: The CDK4/6 inhibitor dalpiciclib augments the antitumor efficacy of enzalutamide in preclinical models of castration-resistant prostate cancer through inhibition of MCM4-mediated DNA replication
Source: Cell Death Dis. 2026 Jun 5;17(1):703. doi: 10.1038/s41419-026-08959-9 (PMC13454615; doi:10.1038/s41419-026-08959-9)
Supplement: Supplementary file 2 [file 41419_2026_8959_MOESM1_ESM.docx]

**Figure S1**

**Figure S1. CDK6 expression in prostate cancer.**

**A**–**B**, CDK6 mRNA expression levels in prostate cancer patient cohorts from the GSE32571 and GSE32269 datasets. Statistical significance was determined using a two-tailed unpaired Student’s t-test. ns, not significant.

**Figure S2**

**Figure S2. Determination of IC_50_ values for enzalutamide and dalpiciclib in CRPC cell lines.**

**A–D,** 22Rv1 and C4-2B cells were treated with increasing concentrations of enzalutamide (ENZ) or dalpiciclib (DAL) for 48 hrs. Cell viability was assessed using a CCK-8 assay. Dose–response curves were generated, and IC₅₀ values were calculated by nonlinear regression analysis. The IC₅₀ values were 51.72 μM for ENZ and 7.62 μM for DAL in 22Rv1 cells, and 70.53 μM for ENZ and 3.73 μM for DAL in C4-2B cells. Data represent the mean ± SD from three independent experiments.

**Figure S3**

**Figure S3. DAL+ENZ combination treatment suppresses cell cycle–related pathways and correlates with CDK6–MCM4 expression in prostate cancer.**

**A,** Dot plot showing the top enriched KEGG pathways in the DAL+ENZ treatment group compared with the DMSO control. **B,** Gene set enrichment analysis (GSEA) of Hallmark gene sets comparing the DAL+ENZ combination treatment group with the control group. The top 20 pathways ranked by |NES| are shown. **C,** Correlation analysis between CDK6 and MCM4 gene expression in the TCGA-PRAD cohort using GEPIA.

**Figure S4**

**Figure S4. Consistent inhibitory effects of different CDK4/6 inhibitors in combination with enzalutamide in CRPC cells.**

**A–B**, CRPC cells were treated with vehicle control, ENZ, palbociclib (PAL), ribociclib (RIB), or their combinations (PAL+ENZ and RIB+ENZ) for 48 hrs. Protein expression levels of RB, phosphorylated RB (p-RB), MCM4, and E2F1 were analyzed by Western blotting. Compared with monotherapy, combination treatment with CDK4/6 inhibitors and ENZ markedly reduced the expression of p-RB, MCM4, and E2F1, indicating enhanced suppression of the CDK4/6–RB–E2F signaling pathway and DNA replication. These results suggest that the synergistic inhibitory effect with ENZ is consistent across different CDK4/6 inhibitors.

**Figure S5**

**Figure S5. Validation of CDK4, CDK6, and AR knockdown efficiency in CRPC cells.**

**A, Relative mRNA expression levels of CDK4, CDK6, and AR in 22Rv1 and C4-2B cells were measured by quantitative real-time PCR (qPCR) 48 hrs after transfection. Data are presented as the mean ± SD from three independent experiments. Statistical significance was determined by one-way ANOVA followed by Tukey’s multiple comparisons test. **p* < 0.05, ***p* < 0.01, ****p* < 0.001 vs. siNC. B, Protein expression levels of CDK4, CDK6, and AR were analyzed by Western blotting 72 hrs after transfection. Among the tested siRNAs, siCDK4-1, siCDK6-1, and siAR-2 showed the highest knockdown efficiency at both mRNA and protein levels and were therefore selected for subsequent experiments.**

**Figure S6**

**Figure S6. Effects of AR or CDK4/6 silencing on ENZ- and DAL-mediated regulation of RB phosphorylation and DNA replication–associated proteins in CRPC cells.**

**A,** C4-2B cells transfected with control or AR-targeting siRNA were treated with DMSO, ENZ (35 μM), DAL (2 μM), or their combination for 48 hrs. Proteins associated with AR signaling, RB phosphorylation, and DNA replication were analyzed by Western blotting. **B,** C4-2B cells transfected with control or CDK4/6-targeting siRNA were treated with DMSO, ENZ (35 μM), DAL (2 μM), or their combination for 48 hrs. Proteins associated with AR signaling, RB phosphorylation, and DNA replication were analyzed by Western blotting.

**Figure S7**

**Figure S7. ENZ and DAL exhibit synergistic antitumor effects in the C4-2B CRPC xenograft model.**

**A,** Representative images of C4-2B CRPC xenograft tumors harvested from each group at the experimental endpoint (day 28). **B**, Tumor growth curves presented as mean ± SD. **C,** Tumor weights at the endpoint presented as mean ± SD. **D,** Body weight curves of mice during the treatment period. **E,** Normalized tumor weight ratio. Tumor weights were normalized to the terminal body weight of mice in each group (n = 6). Data are shown as mean ± SD. Statistical significance was ascertained by one-way ANOVA with Tukey’s multiple comparisons test (B, C, D, E). **p* < 0.05, ***p* < 0.01, ****p* < 0.001.
