# Supplementary material for: The CDK4/6 inhibitor dalpiciclib augments the antitumor efficacy of enzalutamide in preclinical models of castration-resistant prostate cancer through inhibition of MCM4-mediated DNA replication
Source: Cell Death Dis. 2026 Jun 5;17(1):703. doi: 10.1038/s41419-026-08959-9 (PMC13454615; doi:10.1038/s41419-026-08959-9)
Supplement: Supplementary file 3 [file 41419_2026_8959_MOESM2_ESM.docx]

**Supplementary Tables S1-S3**

**Supplementary Table S1. Antibodies used in this study for Western blot analysis.**

| **Antibody** | **Target Antigen** | | **Host** | | **Type** | **Manufacturer** | **Cat. No.** | **Dilution (WB)** |
| --- | --- | --- | --- | --- | --- | --- | --- | --- |
| Primary Antibodies | | GAPDH | Rabbit | Monoclonal | | Cell signaling technology | #2118S | 1:5000 |
|  |  | Caspase 3 | Rabbit | Polyclonal | | Proteintech | 19677-1-AP | 1:1000 |
|  |  | Cleaved Caspase-3 | Rabbit | Monoclonal | | Cell signaling technology | #9664T | 1:1000 |
|  |  | BCL-2 | Rabbit | Polyclonal | | Proteintech | 12789-1-AP | 1:1000 |
|  |  | Bax | Rabbit | Monoclonal | | Cell signaling technology | #5023 | 1:1000 |
|  |  | E2F1 | Mouse | Monoclonal | | Proteintech | 66515-1-lg | 1:1000 |
|  |  | Rb | Mouse | Monoclonal | | Cell signaling technology | #9309T | 1:1000 |
|  |  | Phospho-Rb (Ser807/811) | Rabbit | Monoclonal | | Cell signaling technology | #8516T | 1:1000 |
|  |  | N-cadherin | Rabbit | Monoclonal | | Cell signaling technology | #13116T | 1:1000 |
|  |  | E-cadherin | Rabbit | Monoclonal | | Cell signaling technology | #3195T | 1:1000 |
|  |  | MCM4 | Rabbit | Polyclonal | | Proteintech | 13043-1-AP | 1:1000 |
|  |  | Akt | Rabbit | Monoclonal | | Cell signaling technology | #4691T | 1:1000 |
|  |  | Phospho-Akt (Ser473) | Rabbit | Monoclonal | | Cell signaling technology | #4060T | 1:1000 |
|  |  | PI3K p85 | Rabbit | Monoclonal | | Cell signaling technology | #4257T | 1:1000 |
|  |  | Phospho-PI3K p85 (Tyr458) | Rabbit | Polyclonal | | Cell signaling technology | #4288T | 1:1000 |
|  |  | MCL1 | Rabbit | Polyclonal | | Proteintech | 16225-1-AP | 1:1000 |
|  |  | BCL2L1 | Rabbit | Polyclonal | | Proteintech | 10783-1-AP | 1:1000 |
|  |  | BAK | Rabbit | Polyclonal | | Proteintech | 29552-1-AP | 1:1000 |
| Secondary Antibodies | | HRP-conjugated Goat Anti-Rabbit IgG(H+L) | Goat |  | | Proteintech | SA00001-2 | 1:5000 |
|  |  | HRP-conjugated Goat Anti-Mouse IgG(H+L) | Goat |  | | Proteintech | SA00001-1 | 1:5000 |

**Supplementary Table S2. Primers used for qRT-PCR in this study.**

| **Gene Symbol** | **Forward Primer (5' - 3')** | **Reverse Primer (5' - 3')** |
| --- | --- | --- |
| MCM4 | GACGTAGAGGCGAGGATTCC | GCTGGGAGTGCCGTATGTC |
| AR | CCAGGGACCATGTTTTGCC | CGAAGACGACAAGATGGACAA |
| CDK4 | ATGGCTACCTCTCGATATGAGC | CATTGGGGACTCTCACACTCT |
| CDK6 | GCTGACCAGCAGTACGAATG | GCACACATCAAACAACCTGACC |
| β-actin | TCCCTGGAGAAGAGCTACGA | TGAAGGTAGTTTCGTGGATGC |

**Supplementary Table S3. siRNA sequences used in this study.**

| **Gene** | **siRNA No.** | **siRNA sequence (5’-3’)** |
| --- | --- | --- |
| AR | si#1 | GGAGCUUUCCAGAAUCUGUTT |
|  | si#2 | GUCGCGACUACUACAACUUTT |
|  | si#3 | CCAAUGUCAACUCCAGGAUTT |
| CDK4 | si#1 | GAGGCCUAGAUUUCCUUCATT |
|  | si#2 | CAGUUCGUGAGGUGGCUUUTT |
|  | si#3 | GACUGGCCUCGAGAUGUAUTT |
| CDK6 | si#1 | CCACUGAAACCAUAAAGGATT |
|  | si#2 | GAAGAAGACUGGCCUAGAGTT |
|  | si#3 | GUCUCACCCAUACUUCCAGTT |
